# Specificity Proteins (SP) and Krüppel-like Factors (KLF) in Liver Physiology and Pathology

**DOI:** 10.3390/ijms24054682

**Published:** 2023-02-28

**Authors:** Veera Ganesh Yerra, Konstantinos Drosatos

**Affiliations:** Metabolic Biology Laboratory, Cardiovascular Center, Department of Pharmacology and Systems Physiology, University of Cincinnati College of Medicine, Cincinnati, OH 45267, USA

**Keywords:** Krüppel-like factors, specificity protein, liver physiology, hepatic diseases, hepatocyte, transcriptional regulation

## Abstract

The liver acts as a central hub that controls several essential physiological processes ranging from metabolism to detoxification of xenobiotics. At the cellular level, these pleiotropic functions are facilitated through transcriptional regulation in hepatocytes. Defects in hepatocyte function and its transcriptional regulatory mechanisms have a detrimental influence on liver function leading to the development of hepatic diseases. In recent years, increased intake of alcohol and western diet also resulted in a significantly increasing number of people predisposed to the incidence of hepatic diseases. Liver diseases constitute one of the serious contributors to global deaths, constituting the cause of approximately two million deaths worldwide. Understanding hepatocyte transcriptional mechanisms and gene regulation is essential to delineate pathophysiology during disease progression. The current review summarizes the contribution of a family of zinc finger family transcription factors, named specificity protein (SP) and Krüppel-like factors (KLF), in physiological hepatocyte functions, as well as how they are involved in the onset and development of hepatic diseases.

## 1. Introduction

**Figure 1:**
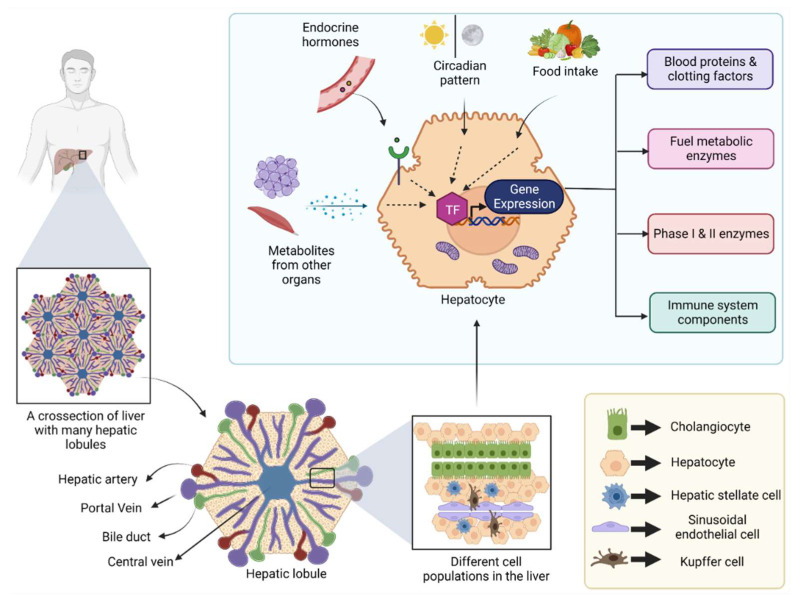
Figure showing different cell populations in the liver and the transcriptional regulation in hepatocytes: Human liver is divided into four lobes, and each lobe contains many hepatic lobules. Each hepatic lobule contains different types of epithelial cells, endothelial cells, and immune cells. Hepatocyte is a specialized parenchymal epithelial cell that can produce several different blood proteins, clotting factors and metabolic enzymes, etc. The gene transcription in hepatocytes is influenced by external stimuli, such as endocrine hormones, and metabolites from other metabolically active organs, food intake, and the day/night cycle. (TF: Transcription factor).

The liver is a large internal organ in the body, which bears remarkable regeneration ability and performs a diverse array of biological functions [1]. In addition to its most known role in systemic energy homeostasis, other functions, such as removal of toxins from the blood, synthesis of various blood proteins, vitamin and mineral metabolism, and immune system regulation, are also controlled by the liver [1]. Located below the diaphragm, the liver is anatomically divided into four lobes which in turn comprise thousands of small lobules (hepatic lobules) [2]. These lobules connect to the small ducts that ultimately merge to form a hepatic duct that carries bile to the gall bladder [2]. Cellular characterization of the liver revealed the presence of several different cell populations, such as hepatic stellate cells, liver macrophages, cholangiocytes, and sinusoidal endothelial cells, along with a major population (80%) of specialized parenchymal epithelial cells termed hepatocytes [3] (Figure 1). 

The liver has an unusual blood supply in the sense that it receives both oxygenated and deoxygenated blood at the same time from both the hepatic artery (accounts for 20–30% of blood supply) and the portal vein (accounts for 70–80% of blood), respectively [4]. The hepatic portal circulation allows deoxygenated blood to enter the liver first, where the toxic substances are cleared and circulated back to the rest of the body. Thus, the liver acts as a guard between the digestive system and the rest of the body. Further, the liver serves as a home for the production of many drug-metabolizing enzymes that helps in detoxification and safe elimination of drugs and xenobiotics. Xenobiotics are foreign chemical substances found within the body that are not normally generated or expected to be present within the organism [5]. Its involvement in xenobiotic metabolism and the direct exposure to the toxins that are ingested through the oral route and end up in the portal circulation makes the liver susceptible to injury and consequent impairment in its function [6]. In fact, drug-induced hepatotoxicity is the most common reason for the withdrawal of marketed drugs [7]. 

Apart from drug or chemical-induced hepatotoxicity, various diseases have been associated with hepatic malfunction, the causes of which range from genetics to infections and impaired metabolism [8]. Chronic liver failure or end-stage liver disease (ESLD) constitutes the common denominator for most of these diseases. Liver cirrhosis is the main culprit of liver failure, which causes healthy liver tissue to be replaced by fibrous scar tissue along with parenchymal nodules and excessive matrix cross linking [9]. Liver cirrhosis was the 11th leading cause of death and 15th leading cause of morbidity in 2016 [10]. Liver cirrhosis, along with viral hepatitis and hepatocellular carcinoma (HCC), contributes to approximately 2 million deaths per year worldwide, which accounts for 3.5% of total global deaths [11]. These are underestimated numbers as accurate data regarding cause-specific mortality is not available from some parts of the world [11]. In addition, increasing trends in obesity incidence and alcohol consumption in many parts of the world further contribute to the risk for liver cirrhosis and ESLD incidence. Despite some breakthroughs in hepatic research, the available therapies to manage cirrhosis and non-alcoholic steatohepatitis (NASH) remain limited, refs. [12,13] underlying the importance of a more comprehensive approach to understanding pathophysiology and discovering novel targets with potential therapeutic ramifications. 

Gene expression in the liver relies significantly on external stimulation [14,15] (Figure 1). In addition, cell signaling in hepatocytes is controlled by circulating hormones, such as insulin, glucagon, epinephrine, steroid, and growth hormone, as well as by metabolites released from other metabolically active organs, including adipose tissue and skeletal muscle [16,17]. Further, the circadian pattern and feed/fast cycle influence this signaling pattern [18,19] (Figure 1). At the molecular level, these events are regulated by the activation or deactivation of various transcriptional factors, such as hepatocyte nuclear factors (HNFs), CCAAT-enhancer-binding proteins (C/EBP), vitamin D-binding protein (DBP) [20], carbohydrate response element binding protein (ChREBP), liver X receptor (LXR), sterol regulatory element-binding protein 1 (SREBP-1c), peroxisome proliferator-activated receptor alpha (PPAR-α), farnesoid X receptor (FXR), Forkhead Box O1 (FOXO1), cAMP-response element binding protein (CREB), and the co-transcriptional activator peroxisome proliferator-activated receptor gamma coactivator 1-alpha (PGC-1α) [21]. These factors modulate the expression of specific metabolism-related enzymes that affect either liver-specific or systemic metabolism in response to the body’s energetic demands [21]. Various hepatic diseases have been associated with altered expression or post-translational modifications of these transcriptional factors [22,23]. Therefore, targeted modulation of these transcriptional signaling events serves as a drug discovery platform for the management of hepatic diseases [24,25].

Zinc finger transcription factors are a sub-class of transcription factors that share a common DNA binding domain in their structure and are known to influence the transcription of several genes important for cell growth, differentiation, survival, and metabolism in the liver and other tissues. Specificity Protein (SP) 1 and Krüppel-like Factors (KLF) are zinc finger domain-containing transcription factors that have been extensively studied for their role and involvement in several different cell functions [26]. However, a comprehensive review covering the role of these two protein families in relation to the hepatobiliary system has not been published. In this review, we provide a summary of the role of SP and KLF transcription factors during embryonic development of the liver, as well as in health and hepatic injury or disease.

## 2. SP and KLF Transcription Factors

Transcription factors are trans-acting DNA binding elements that can regulate the transcription of specific genes in association with other partners of the transcriptional machinery [20]. Three structural domains are critical for the function of a transcriptional factor. These are the DNA binding domain, the nuclear localization signal domain, and the regulatory domain [27]. SP and KLF transcription factors share a highly conserved carboxyl-terminal DNA binding domain with a sequence similarity of more than 65% among the members. This domain is characterized by three in-tandem repeats of Cys_2_ His_2_ zinc finger motifs [26]. These motifs allow binding to the GC-rich proximal promoter regions or CACC elements (GT boxes) in the promoter regions of several genes [26]. The classical sequence of a C2H2 zinc finger motif is #-X-C-X(1-5)-C-X3-#-X5-#-X2-H-X(3-6)-[H/C], where C and H represent cysteine and histidine and X represents any amino acid. The numbers represent the residues separating the flanking amino acids. The “#” mark indicates the amino acids that are critical for the proper folding of zinc fingers [28]. Whereas the carboxyl-terminal consists mostly of the conserved DNA-binding domain, the amino-terminal is highly variable among different members of the KLF and SP family. This variability in the amino-terminal domain underlies the plasticity in their interaction with various transcriptional coactivators and corepressors and their consequent effects on gene expression [26].

So far, a total of 18 members of the KLF family and 9 members of the SP family transcription factors have been identified in mammals [29,30]. Based on the phylogenetic and structural architecture, KLFs are divided into three different subfamilies consisting of:

- KLF1, KLF2, KLF 4, KLF5, KLF6, and KLF7,
- KLF3, KLF8, and KLF12 group,
- KLF9, KLF10, KLF11, KLF13, KLF14, and KLF16 group [31].

KLF15 and KLF17 are not classified into these groups as they are more distantly related on the phylogenetic scale [31]. The structural homologies in these members also correlate in their functional similarities due to the similarity in the amino-terminal interaction domain among related members [31]. For instance, KLFs in group 1 act mostly as transcriptional repressors through interaction with C-terminal binding protein (CtBP), whereas KLFs in group 2 act as transcriptional activators. KLFs in group 3 also act as repressors through interaction with co-transcriptional repressors such as Sin3a [31].

Based on structural similarities, SPs are further divided into two sub-families:

- SP1, SP2, SP3, and SP4,
- SP5, SP6, SP7, SP8, and SP9 [32].

A button head (Btd) box domain that is comprised of 10 amino acids and lies prior to the carboxyl-terminal DNA-binding domain differentiates SP transcription factors from KLFs [30]. Studies in Drosophila have shown that this domain is involved in embryonic head segmentation and limb development [33,34]. In mammals, it was also found to be important during embryonic development [35]. More recently, it was shown that the Btd domain of SP1 interacts with SREBP1a and controls the expression of the low-density lipoprotein (LDL) receptor [36].

Gain- and loss-of-function studies have identified that SP1-like or KLF family proteins are involved in the growth regulatory functions of many tissues [37]. Whereas some members are important for many tissues, some other members act in a cell-specific manner [38,39]. With respect to the liver, SP/KLF transcription factors have a documented role in controlling the expression of various genes important for liver function under normal conditions, and their dysregulation has been implicated in developing hepatic pathologies. A detailed summary of the role played by these transcription factors in hepatic physiology/pathology is lacking, and hence the current manuscript is expected to fill in this void and serve as a resource document for those who may be interested in studying them in more detail. 

## 3. SP and KLF Transcription Factors in a Healthy Liver

### 3.1. Embryonic Liver Development

Embryonic liver development starts as early as day E8 after gastrulation and demarcation of germ layers. The liver originates from the ventral foregut part of the embryonic endoderm, and the primary morphological development of the liver occurs in the form of a hepatic diverticulum by E9, which later develops into a liver bud by E10 [40]. The liver bud later undergoes rapid development during E10–E15, when it is vascularized and innervated with hematopoietic progenitors [40]. The development and differentiation of hepatoblasts into hepatocytes and biliary epithelial cells continue in the post-natal period. While most of the liver originates from the endoderm, the stellate cells and hepatic fibroblasts, and endothelial cells originate from the embryonic mesoderm [41]. It is believed that there is a series of reciprocal interactions between these two layers that control hepatic development [40]. While studies on the molecular signaling events that underlie liver development have implicated the transcription factors Forkhead Box A2 (Foxa2), GATA binding factor 4 (Gata-4), GATA binding factor 6 (Gata-6), Hematopoietically expressed homeobox (Hhex), ref. [42] Hepatocyte Nuclear Factor 4 (HNF4), C/EBP, Oncostatin M (OSM), Hepatocyte growth factor (HGF), and Proto-oncogene Wnt (Wnt) [42], the importance of SP and KLF factors in liver development is relatively understudied. The significance of SP transcription factors in embryonic hepatic development and fetal liver-mediated erythropoiesis was demonstrated in a seminal study where authors explored the developmental defects of *Sp1*^+/−^*Sp3*^+/−^ heterozygous mice [43]. The resultant embryos displayed significant lethality, and no mice were obtained by the stage of weaning associated with developmental defects. The viable embryos of *Sp1*^+/−^*Sp3*^+/−^ mice at E16.5 and E14.5 days show diminished fetal liver weight and erythroid progenitors (CD71^med-low^Ter119^high^) compared to their wild-type counterparts [43]. Further, defects in fetal liver-mediated erythropoiesis were also evident via reduced erythroid colony-forming units (CFU-Es) and colony-forming units from megakaryocyte progenitors (CFU-MKs) in *Sp1*^+/−^*Sp3*^+/−^ E14.5 embryos [43].

The early evidence of KLF's importance for the embryonic hepatic function came from the murine liver development studies in which Erythroid Krüppel-like factor (EKLF) transcription factor was shown to regulate the fetal liver-mediated erythropoiesis as *EKLF1*^−/−^ mice displayed fetal anemia [44]. Later, the importance of KLF6 to embryonic hematopoiesis and liver development was demonstrated in mice and embryonic stem cells. Apart from markedly reduced hematopoiesis and yolk sac vascularization, *Klf6*^−/−^ embryos failed to develop a clearly defined liver [45]. These in vivo observations were also supported by in vitro studies showing reduced hematopoietic differentiation in stem cells isolated from these embryos [45]. However, this study has failed to separate the role of KLF6 in hepatogenesis from early hematopoiesis. Later studies in zebrafish have clearly shown that *klf6* (*copeb* in Zebra fish) deficiency failed to yield hepatic outgrowth, highlighting the importance of KLF6 in the early stage of endoderm maturation to the liver. Studies in mouse embryonic stem cells showed that *Klf6* ablation reduces the expression of endoderm marker genes such as *Hnf3β*, *Gata4*, *Sox17*, and *CxCr4,* but overexpression of *Klf6* corrects their expression [46]. Another study detected KLF13 expression in embryonic liver vasculature at E14.5, although its exact function is not defined [47]. KLF15 has been recently shown to regulate the development of mature hepatocyte-like cells from embryonic hepatoblasts isolated from mouse liver. Similarly, retrovirus-mediated overexpression of KLF15 in iPSC-derived hepatoblasts induced expression of the hepatocyte markers *Tat*, *Cps1*, *Cyp1a2,* and *Cyp2e1* implicating the importance of KLF15 for the hepatocyte-specific lineage [48]. 

### 3.2. Glucose Metabolism

The liver controls blood glucose levels during feeding and fasting cycles, in concert with other metabolically active organs such as the skeletal muscle and adipose tissue. This is, in turn, facilitated by the endocrine regulation of insulin, glucagon, epinephrine, and growth hormone [49]. During feeding, glucose enters hepatocytes through Glucose transporter 2 (GLUT2) and is stored in the form of glycogen [49]. Further, glucose is also broken down into pyruvate during glycolysis, and pyruvate is further oxidized into acetyl coA, which is either oxidized by the Krebs cycle and oxidative phosphorylation to yield ATP or is used for the synthesis of fatty acids. During fasting, the glycogen reserves in the liver undergo glycogenolysis to produce glucose. When glycogen reserves are depleted, the liver generates glucose from non-carbohydrate sources (lactate, glycerol, alanine) through gluconeogenesis [49].

**Figure 2:**
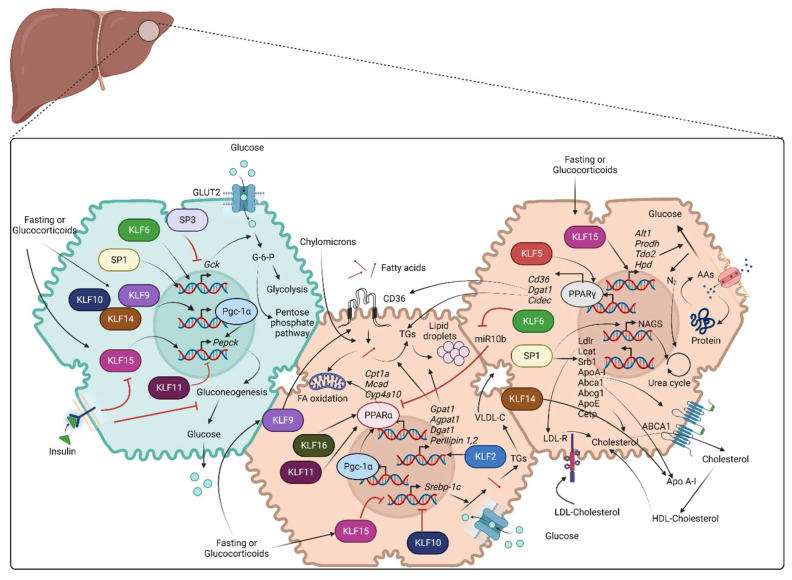
Role of SP/KLF transcription factors in metabolism: Green shade represents glucose metabolism, and brown shade represents lipid and amino acid metabolism. Glucose metabolism: Glucose enters hepatocytes through GLUT2 and is converted to glucose-6-phosphate with the contribution of the Gck enzyme. Glucose-6-phosphate further participates in glycolysis or pentose phosphate pathway. SP1 and KLF6 stimulate the transcription of *Gck*. Fasting or glucocorticoid stimulation induces KLF9, KLF10, KLF14, and KLF15. KLF9, KLF10, and KLF14 stimulate transcription of *Ppargc1a*, whereas KLF15 along with PGC-1α, promotes the transcription of *Pepck* that encodes the rate-limiting step of gluconeogenesis, leading to increased hepatic glucose production. KLF11 suppresses gluconeogenesis via transcriptional repression of *Pepck* and *Ppargc1a*. Lipid metabolism: Fatty acid uptake is mediated by CD36 and is followed by TG storage in lipid droplets or β-oxidation in mitochondria. Hepatocytes carry out de novo lipogenesis from pyruvate and the synthesis of cholesterol that is secreted as a component of VLDL or HDL particles. SP1 stimulates the transcription of several genes that are important for cholesterol metabolism, including *LDLR*, *LCAT*, *SRB1*, *APOA-I*, *ABCA1*, *ABCG1*, *APOE*, and *CETP*. KLF14 stimulates the transcription of *APOA-I*. KLF2 and KLF9 promote lipid uptake via transcription of *Cd36*. KLF2 promotes the transcription of enzymes of TG synthesis and lipid storage (*Gpat1*, *Agpat1*, *Dgat1*, *Perilipin 1*, *Perilipin 2*). KLF11 and KLF16 promote β-oxidation of fatty acids by facilitating PPARα-mediated transcription of *Cpt1a*, *Mcad*, *Cyp4a10*, and *Cyp4a14*. KLF6 indirectly stimulates PPARα-mediated transcription of *Trb3* by suppressing transcription of miR10b. KLF10 and KLF15 suppress SREBP-1c-mediated hepatic lipogenesis. KLF5 promotes the transcription of genes for lipid uptake and metabolism (*Cd36*, *Dgat1*, *Cidec*) through PPARγ stimulation. Amino acid/protein metabolism: Hepatocytes obtain circulating AAs through transporters or from degraded protein/polypeptides and use their carbon backbone for gluconeogenesis. Ammonia released from this reaction is toxic and enters the urea cycle. SP1 enhances the transcriptional induction of NAGS, which is essential for the urea cycle. On the other hand, KLF15 encodes transcription of the enzymes *Alt1*, *Prodh*, *Tdo2*, and *Hpd,* which are essential for AA metabolism and, eventually, gluconeogenesis (AA: Amino acids, Abca1: ATP binding cassette subfamily A member 1, Abcg1: ATP binding cassette subfamily G member 1, Agpat1: 1-acyl-sn-glycerol-3-phosphate acyltransferase alpha, Alt1: Alanine aminotransaminase 1, Apo A-I: Apolipoprotein A-I, ApoE: Apolipoprotein E, Cetp: Cholesteryl ester transfer protein, Cidec: Cell death-inducing DFFA-like effector C, Cpt1a: Carnitine palmitoyltransferase 1A, Cyp4a10: Murine cytochrome P450, family 4, subfamily a, polypeptide 10, Dgat1: Diacylglycerol O-Acyltransferase 1, FA: Fatty acids, Gck: Glucokinase, GLUT2: Glucose transporter 2, Gpat1: Glycerol-3-phosphate acyltransferase 1, HDL: High density lipoprotein, Hpd: 4-Hydroxyphenylpyruvate dioxygenase, KLFs: Krupple like family transcription factors, Lcat: Lecithin-cholesterol acyltransferase, Ldlr: Low-density lipoprotein receptor, Mcad: Medium-chain acyl-CoA dehydrogenase, NAGS: N-acetyl glutamate synthase, PGC-1α: Peroxisome proliferator-activated receptor-gamma coactivator-1alpha, PPARα: Peroxisome proliferator-activated receptor alpha, PPARγ: Peroxisome proliferator- activated receptor gamma, Prodh: Proline dehydrogenase, SP: Specificity protein family transcription factors, Srb1: Scavenger receptor class B type 1, Srebp-1c: Sterol regulatory element-binding protein 1, Tdo2: Tryptophan 2,3-dioxygenase, TG: Triglycerides, VLDL: Very low density lipoprotein).

Multiple transcription factors such as FOXOs, HNFs, CREB, and CEBPα/β have been characterized to regulate various enzymes involved in glycolysis, glycogenolysis, and gluconeogenesis [50,51,52]. The role of KLF factors in these critical glucose-regulating pathways has been studied in animal models of diet-induced obesity (DIO) and in cell culture studies. The respective role of SPs has been studied less thoroughly. Figure 2 provides an overview of the role of various SP/KLF transcription factors in glucose metabolism along with lipid and protein metabolism.

The role of KLF3 in liver biology, although studied, is still unclear. For instance, *Klf3*^−/−^ mice displayed lower liver weight than wild-type mice fed on both a chow diet and a high-fat diet, although the reason for this reduction is not explained. *Klf3*^−/−^ mice also displayed better glucose tolerance with HFD, which was attributed to de-repression (activation) of Family with sequence similarity 132, member A (Fam132a) (also termed adipolin) in most of the glucose-responsive tissues (excluding liver) [53]. 

KLF6 has also been studied for its role in hepatic insulin resistance and glucose metabolism. Studies in murine hepatocytes indicate that KLF6 binds to the promoter of Glucokinase (*Gck*) and increases its expression, leading to hepatic insulin resistance and the development of non-alcoholic fatty liver disease (NAFLD) [54]. 

KLF9 contributes to hepatic gluconeogenesis in response to dexamethasone administration and fasting [55]. These findings were confirmed by KLF9 gain- and loss-of-function studies in hepatocytes that demonstrated its importance in mediating glucocorticoid-induced hepatic glucose production. Further, molecular examination of the cell signaling pathways unraveled the importance of PGC-1α downstream of KLF9 [55]. Specifically, both global and hepatocyte-specific *Klf9* KO mice (Alb-*Klf9*^−/−^) displayed hypoglycemia upon fasting because of suppressed hepatic gluconeogenesis. PGC-1α overexpression in Alb-*Klf9*^−/−^ mice rescued fasting-induced hypoglycemia [55]. Further studies in type-2 diabetes mouse models (db/db and ob/ob) showed increased KLF9 expression in their livers. Accordingly, knocking out *Klf9* specifically from hepatocytes improved glucose tolerance and reduced hepatic gluconeogenesis [55]. Studies in zebrafish pointed out the importance of KLF9 towards glycolysis as KLF9 has been shown to inhibit glycolytic flux and promote the pentose phosphate pathway in response to stress in these embryos [56]. 

Hepatic gluconeogenesis is also controlled by KLF10, the expression of which is elevated in the livers of diabetic, obese mice, and DIO mice. Adenovirus-mediated constitutive expression of KLF10 in primary mouse hepatocytes increases the expression of *Ppargc1a*, *Pck1,* and *G6pc*. On the other hand, the knockdown of *Klf10* reduces forskolin-induced gluconeogenesis-related gene expression [57]. Similarly, in vivo silencing of KLF10 with Ad-shKLF10 administration attenuates glucose intolerance in db/db and DIO mice via inhibition of the hepatic expression of gluconeogenesis-related genes [57]. It has also been observed by K Lizuka et al. that glucose-induced transcription factor, ChREBP, controls the expression of *Klf10* in hepatocytes by direct promoter binding, and this crosstalk between these two transcription factors might be an important event for ChREBP-mediated hepatic lipogenesis [58].

Similarly, hepatic expression of KLF14 is induced in healthy mice upon fasting and in db/db mice. Adenovirus-mediated overexpression of KLF14 in mouse primary hepatocytes stimulates hepatic glucose production through induction of PGC-1α [59]. Accordingly, the knockdown of KLF14 results in the opposite effect [59]. In support of these in vitro findings, overexpression of KLF14 in healthy mice lowered glucose tolerance, and knockdown of KLF14 in db/db mice attenuated glucose intolerance [59].

As discussed above, KLF9, KLF10, and KLF14 are induced in response to fasting in the liver and stimulates hepatic gluconeogenesis by indirectly promoting the transcription of rate-limiting enzyme, Phosphoenolpyruvate carboxykinase (Pepck) through the transcriptional activation of *Ppargc1a* [60]. In contrast to these factors, KLF11 has the opposite effect on gluconeogenesis by suppressing the expression of *Pepck* [61]. In primary hepatocytes, Ad-KLF11 did not affect gluconeogenesis under basal conditions, but it significantly inhibited the expression of PGC-1α, PEPCK-C, and G6PC upon treatment with forskolin and dexamethasone, which mimic the actions of glucagon and glucocorticoid, respectively [61]. 

One of the most studied KLF isoforms in hepatic glucose metabolism is KLF15. KLF15 was initially studied for its role in gluconeogenesis [62]. Studies in cultured hepatocytes suggest that KLF15 increases PEPCK expression [63]. Further, it was shown that dexamethasone and non-hydrolyzing (stable) analog of 3′,5′-cyclic adenosine monophosphate (cAMP) (to mimic glucagon action) induce hepatic KLF15 expression, whereas insulin elicits an inhibitory effect through PI3K signaling [63]. In support of these observations, mice with targeted global deletion of KLF15 developed fasting-induced hypoglycemia due to impaired amino acid degradation and reduced availability of metabolites for glucose production [62]. These mice displayed reduced hepatic expression of alanine aminotransferase (ALT), which converts alanine to pyruvate to enhance hepatic glucose production. Consequently, supplementation of pyruvate to *Klf15*^−/−^ mice attenuated fasting hypoglycemia [62]. KLF15 controls the expression of various gluconeogenesis and amino-acid-degrading enzymes in coordination with PGC-1α. Hepatic ablation of KLF15 in diabetic mice attenuated the expression of these genes and reduced glucose production [64]. Further, it was also shown that metformin suppresses hepatic expression of KLF15 and its target enzymes of gluconeogenesis and amino acid-degradation in mouse primary hepatocytes and diabetic mice [64]. 

In contrast to the clear-cut roles established for KLF factors, the role played by SP transcriptional factors in hepatic glucose metabolism is mostly indirect through regulation of other proteins, such as Fibroblast growth factor 21 (FGF21), high mobility group A1, leptin, that influence systemic glucose metabolism [65,66,67]. Analysis of murine *Fgf21* proximal promoter region identified important SP1 binding sites (located between −46 and −38) that are essential for its transcription [66]. Although not in mammals, SP1 and SP3 have been found to regulate the expression of the *Gck* gene in gilthead sea bream (*Sparus aurata*). Electrophoretic mobility shift experiments in HepG2 cells showed that SP1 transactivates the *Gck* promoter while SP3 represses it by competitive binding [68]. However, SP1 nuclear localization and post-translational changes were found to be significantly influenced by Insulin [69], and hence there seems to be some possible role for SP1 in insulin-mediated hepatic effects, which are not clearly established at this moment. SP1 has also been identified to possibly regulate the F-type specific mRNA transcription of a bifunctional enzyme, 6-phosphofructo-2-kinase (PFK2)/fructose-2,6-bisphosphatase (FBPase2), that catalyzes the synthesis and degradation of fructose-2,6-bisphosphate, a stimulator of glycolysis in rat hepatoma cells [70]. The expression of this isoform is limited to fetal rat liver and some other tissues, and if similar mechanisms operate in the transcription of L-type (liver-specific), isoform expression is not clear. 

### 3.3. Fatty Acid and Cholesterol Metabolism

Lipids—mainly fatty acids and triglycerides (TG)—constitute a major metabolic fuel for the body, and the liver plays a central role in their homeostasis. The fate of lipids in the liver can be easily studied by classifying them into four different aspects: (i) acquisition of lipids (de novo lipogenesis, uptake of TGs and FAs from circulation either in the form of free fatty acids or as components of high-density lipoprotein (HDL), low-density lipoproteins (LDL), and chylomicron remnants) (ii) lipid storage and deposition as TG in the liver (iii) release of lipids via secretion of very low-density lipoproteins (VLDL), (iv) lipid consumption (β-oxidation of fatty acids, secretion of ketone bodies and cholesterol synthesis) [71]. Hepatocytes derive fatty acids from chylomicron particles derived from the gut or from circulating non-esterified fatty acids (NEFA) derived from adipose tissue. In addition to these routes, hepatocytes can also synthesize fatty acids from excess glucose and lipolysis of stored TG. Fatty acids obtained via these routes are utilized for various important aspects such as the synthesis of phospholipids and cholesterol, energy metabolism by FA oxidation, storage as lipid droplets, and secretion as VLDL particles [49]. Both KLFs and SPs have been studied for their influence on lipid metabolism in both hepatic and non-hepatic cells (Figure 2). 

KLF2 expression is increased in the liver of ob/ob mice and wild-type mice fed on HFD. Adenovirus-mediated overexpression of KLF2 in healthy mice promotes hepatic steatosis through increased *Cd36* gene expression, TG synthesis (*Gpat1*, *Agpat1*, *Dgat1*), and lipid storage (*Perilipin 1*, *Perilipin 2*, *Cidea*) gene expression [72]. On the other hand, shRNA-mediated suppression of KLF2 reduces liver weight and TG deposition of ob/ob mice in a CD36-dependent manner. Chromatin immunoprecipitation and site-directed mutagenesis studies confirmed that CD36 is a direct transcriptional target of KLF2 [72]. 

In contrast to the direct role of KLF2 in hepatocyte biology, KLF4 indirectly influences the hepatocyte genetic program through M2 polarization in Kupffer cells and release of IL-10, which prevents the development of HFD-induced hepatic steatosis and NASH pathogenesis [73]. Mechanistic studies identified retinoic acid-related orphan receptor α (RORα) as a key regulator of M1/M2 polarization, which induces M2 maturation through induction of KLF4 [73]. KLF4 has also been identified to increase the transcription of HDL receptor scavenger receptor class B type I (SR-BI) by direct promoter binding in response to high-density lipoproteins treatment in THP-1 cells [74].

A surprising role for KLF5 in hepatic lipid metabolism was uncovered while studying the role of ubiquitin ligase, F-box, and WD repeat domain-containing 7α (Fbw7α) [75]. In vivo knockdown of *Fbw7α* resulted in steatohepatitis in C57BL/6J mice through indirect regulation of peroxisome proliferator-activated receptors γ2 (PPARγ2) and its target genes involved in fatty acid uptake and triglyceride synthesis (fatty acid transporter *Cd36,* diacylglycerol acyltransferase 1 *(Dgat1),* fat specific protein 27 *(Cidec)*) [75]. The same study confirmed that Fbw7α mediates KLF5 degradation, and treatment of hepatocytes isolated from *Klf5* floxed mice with adenovirus expressing Cre recombinase showed reduced PPARγ2 expression, implicating the KLF5-PPARγ2 signaling pathway as the underlying mechanism of Fbw7α-mediated hepatic lipid metabolism effects [75]. 

KLF6 has also been studied for its role in HFD-induced hepatic steatosis in mice. Either global heterozygous mice or hepatocyte-specific *Klf6* knockout mice were protected from HFD-induced fat deposition and liver abnormalities. Similarly, these mice had improved systemic insulin response and attenuated hepatic gluconeogenesis. Mechanistic studies revealed that KLF6 indirectly promotes the PPARα expression at the post-transcriptional level through transcriptional repression of miR10b [76]. Thus, post-transcriptional induction of PPARα and consequent influence on the expression of *Trb3* and *Pepck* expression could account for the detrimental influence of KLF6 on systemic lipid metabolism.

KLF9 is one of the transcriptional factors that has been studied well for its role in hepatic lipid metabolism. An increase in KLF9 expression was observed in HFD-fed mice, and KLF9 genetic ablation from hepatocytes attenuated fatty liver phenotype after HFD [77]. Molecular studies in primary hepatocytes treated with Ad-KLF9 or Ad-shKLF9 indicated that KLF9 directly increases the expression of *Cd36* and increases intracellular TG content, supporting the in vivo observations [77], whereas the above results suggest that KLF9 promotes hepatic lipid deposition under fat-fed conditions, a strikingly contrasting effect of KLF9 on hepatic fatty acid oxidation observed under fasting conditions. This study revealed the induction of KLF9 in response to fasting or dexamethasone treatment and promoted PGC-1α-mediated hepatic fatty acid oxidation [55]. Global and hepatocyte-specific *Klf9* deletion showed normal phenotype under basal conditions but displayed hepatic steatosis and increased TG deposition in the liver when fasted for 24 h, indicating the role of KLF9 in fasting-induced hepatic fatty acid oxidation [55]. In support of these observations, these mice displayed lower levels of PPARα target genes *Mcad*, *Cpt1a*, *Cyp4a10,* and *Cyp4a14* expression, as well as of lipogenesis-related proteins (*Srebp1c*, *Acc,* and *Scd1*) [55]. Finally, adenovirus-mediated overexpression of PGC1α reversed hepatic steatosis in hepatocyte-specific *Klf9* knockout mice, confirming that KLF9-mediated hepatic fatty acid oxidation is predominantly mediated through PGC-1α [55]. So, based on these studies, it is evident that KLF9 has a differential role in hepatic fatty acid metabolism under fasting and fed conditions, promoting the fatty acids for energy utilization during fasting and causing increased uptake and steatohepatitis under fed conditions.

KLF10 is also studied for its role in hepatic lipid metabolism. Hepatocyte-specific *Klf10* knockout mice, when challenged with sugar-sweetened water (SSW) for eight weeks, showed a higher increase in liver weight, TG content, and hepatic steatosis than their wild-type controls. This is associated with higher expression of fatty acid synthase (*Fasn*) and fatty acid elongase (*Elovl6*) and lower expression of acyl-CoA synthetase short-chain family member 2 (*Acss2*) and acetyl-CoA carboxylase beta (*Acacb*), suggesting the role of KLF10 in fatty acid oxidation and prevention of lipogenesis [78]. Surprisingly, KLF10 deletion influenced the expression of various metabolic genes in a circadian-specific manner, meaning that KLF10 regulates the liver fatty acid metabolism according to the day/night cycles. Another study showed that KLF10 depletion from hepatocytes leads to severe liver injury and progression to hepatic fibrosis in response to a high-sugar diet, suggesting defects in lipid metabolism in these mice could contribute to fibrosis [79]. Although it is clear from the above studies that KLF10 controls hepatic fatty acid oxidation, it is unclear if KLF10 also has any role to play in hepatic lipid uptake and TG deposition. This was later disclosed in a study where authors demonstrated increased expression of KLF10 in HFD-fed mice and western diet/carbon tetrachloride (CCL4)-treated mice contributing to increased lipid uptake and steatohepatitis [80]. Molecular studies in mouse primary hepatocytes indicated that KLF10 transcriptionally induces the expression of palmitoyl acyltransferase *zDHHC7* that can cause palmitoylation and membrane localization of CD36, leading to increased fatty acid uptake and TG deposition, explaining the basis for KLF10-mediated lipid uptake [80]. Thus, from the existing literature, it is unclear if KLF10 promotes hepatic lipid deposition or hepatic fatty acid oxidation, as there is evidence supporting both observations. It is possible that the actions of KLF10 on lipid metabolism could be different depending upon the type of meal (carbohydrate-rich vs. fatty meal), promoting fatty acid oxidation in the former vs. promoting fatty acid uptake and TG deposition in the latter condition.

Studies in db/db and HFD-induced obese mice demonstrated the importance of KLF11 in hepatic lipid metabolism [81]. Hepatic expression of KLF11 was reduced in both these models, while overexpression of KLF11 in HFD-fed mice and in db/db mice prevented the development of fatty liver via induction of PPARα-mediated expression of fatty acid oxidation genes, such as *Cpt1a*, *Mcad*, *Cyp4a10,* and *Cyp4a14* [81]. Accordingly, in vivo silencing of KLF11 by administration of Ad-shKLF11 in wild-type C57BL/6J and db/m mice increased liver weight and hepatic TG deposition, suggesting possible impairment of PPARα-mediated fatty acid oxidation [81]. This study thus clearly demonstrated the role of KLF11 in physiologically PPARα-mediated hepatic fatty acid oxidation. 

KLF14 has also been studied for its involvement in liver fatty acid and cholesterol metabolism. Its overexpression in mouse liver increases serum TG levels, and free fatty acid levels correspond to increased PGC-1α-mediated hepatic fatty acid oxidation [59]. Given the opposite functions of Insulin on hepatic fatty acid oxidation and its influence on PGC-1α signaling, this possibly suggests that KLF14 might have some role to play in insulin resistance. On the other hand, another study has shown insulin-sensitizing effects of KLF14 as KLF14 overexpression has been found to increase the phosphorylation of insulin receptor (InsR), insulin receptor substrate-1 (IRS-1), glycogen synthase kinase-3β (GSK-3β), and Akt and enhanced insulin-mediated glucose uptake in Hepa1-6 cells [82]. Guo et al. have shown that hepatocyte-specific KLF14 overexpression increases plasma HDL cholesterol by transcriptional induction of apolipoprotein (ApoA-I), whereas the deletion of KLF14 lowers plasma HDL levels. Pharmacological KLF14 activation by perhexiline increases HDL cholesterol level and cholesterol efflux capacity in an apoA-I-dependent manner [83]. These studies available so far have thus identified KLF14's role in hepatic fatty acid oxidation and insulin-mediated hepatic glucose uptake and transcriptional induction of ApoA-I and thus regulation of HDL-C levels.

KLF16 regulates hepatic lipid metabolism by directly influencing the transcription of *Ppara*. Hepatocyte-specific knockout of KLF16 impairs fatty acid oxidation and aggravates mitochondrial stress, whereas overexpression abrogates these effects [84].

KLF15 has also been studied for its contribution to hepatic lipid metabolism and insulin resistance. D Y Jung et al. have identified that *Klf15*^−/−^ mice have better hepatic insulin sensitivity and attenuated hepatic steatosis in response to HFD [85]. Consistent with reduced hepatic lipid content, *Klf15*^−/−^ livers exhibited reduced mRNA levels of fatty acid transport protein (*Fatp*), fatty acid synthase (*Fasn*), and Apolipoprotein A-IV (*ApoA-IV*), and increased fatty acid oxidation genes such as peroxisome proliferator-activated receptor gamma coactivator-1 alpha (*Ppargc1a*) and carnitine palmitoyl transferase 1a (*Cpt1a*) [85]. However, *Klf15*^−/−^ hepatocytes displayed increased endoplasmic stress (unfolded protein response, UPR) markers and KLF15 overexpression through adenoviral infection in hepatocytes attenuated basal and Tunicamycin induced UPR markers, indicating that KLF15 controls ER stress response. This uncoupling of ER stress and insulin resistance in HFD-fed *Klf15*^−/−^ mice is explained by the possible induction of 5′-AMP-activated protein kinase (AMPK) and inhibition of mTORC1 signaling and stimulation of PGC-1α, leading to increased fatty acid oxidation [85]. In contrast to these earlier findings, where KLF15 has been identified for its pathological role in HFD-induced insulin resistance and hepatic steatosis, under fasting conditions, KLF15 was identified to inhibit hepatic lipogenesis by suppressing the *Srebf1c* transcription [86]. At the molecular level, these events are found to be mediated through interaction with the LXR/RXR complex and recruitment of corepressor RIP140 [86]. These authors have also demonstrated that overexpression of KLF15 in ob/ob mice attenuated *Srebf1* overexpression and reduced hypertriglyceridemia [86]. In addition to these studies, KLF15 has also been studied for its importance in lipid metabolism in skeletal muscle [87,88]. These findings also highlight that skeletal muscle-specific loss of KLF15 increased fat deposition in the liver and WAT under baseline conditions, indicating a global role for KLF15 in systemic lipid metabolism [88]. On the whole, these studies suggest that KLF15 is an important contributor and critical regulator of hepatic lipid metabolism, although more studies need to be done to exactly understand its role under different metabolic conditions. 

Similarly, genes important for cholesterol and lipoprotein metabolism are also found to be modulated by SP family transcription factors. For instance, the human LDL receptor gene promoter is coordinatively activated by SREBP1 and SP1 [89]. In a similar manner, the promoter region of human lecithin: cholesterol acyltransferase (LCAT), which catalyzes the maturation of HDL, revealed the presence of two binding sites for SP transcription factors (SP1 and SP3) [90]. Functional characterization identified that SP1 transactivates the LCAT promoter, whereas SP3 acts as a dose-dependent repressor [90]. The HDL receptor scavenger receptor class B type I (SR-BI) is also induced at the transcriptional level by SP1 and SP3 through direct promoter binding [91]. Further, other genes important for lipoprotein metabolism, such as apoA-I [92,93], ABCA1 [94,95], ABCG1 [96], apo E [97], and CETP [98] genes, were also found to be transcriptionally modulated by SP1. In most of these cases, while SP1 acts as a transcriptional activator of these genes by binding to its consensus sites in the promoter or enhancer region, SP3 mostly opposes their transcription (ABCA1, LCAT, and CETP) by competing with SP1 for binding. Overall, these multiple studies clearly highlight that SP1 and SP3 control the transcriptional expression of several genes important for cholesterol/lipoprotein metabolism under basal conditions. 

### 3.4. Amino Acid and Protein Metabolism

Amino acids are taken up by hepatocytes from the circulation and used unmodified for protein synthesis (albumin and clotting factors), or they are deaminated prior to conversion to metabolic fuels, such as fatty acids (lipogenesis) or glucose (gluconeogenesis) [49]. The amine group released from such a deamination reaction is converted to ammonia, which is very toxic to the body, and hence the liver converts it into urea [99]. Proteins and peptides are taken up by liver cells via receptor-mediated endocytosis and are eventually broken down to amino acids that can be used for protein synthesis or conversion into metabolic fuel. Both SP and KLF family transcription factors regulate the expression of genes that encode proteins for the uptake and metabolism of amino acids in hepatocytes (Figure 2).

SP1 is important for the induction of expression of human N-acetyl glutamate synthase (NAGS), the key enzyme responsible for the production of NAG, which acts as a cofactor for rate-limiting enzyme of the urea cycle, carbamoyl phosphate synthetase 1 (CPS1) [100]. 

KLF15 is one of the transcription factors that was well studied for its role in amino acid catabolism that precedes gluconeogenesis. It has been observed that mice deficient in KLF15 expression have more pronounced fasting-induced hypoglycemia due to defects in the availability of amino acid-catabolizing enzymes, such as alanine aminotransferase 1 (ALT1), proline dehydrogenase (ProDH), tryptophan 2,3-dioxygenase (TDO2) and 4-hydroxyphenylpyruvic acid dioxygenase (HPD) [62]. In particular, the enzyme responsible for the conversion of alanine to pyruvate, ALT1, is reduced by 50% in *Klf15^−/−^* hepatocytes [62]. Later, it was identified that FoxO1 and FoxO3a could directly bind to the promoter region of KLF15 and induce its transcription in response to fasting, which in turn controls the transcription of amino acid metabolizing enzymes *Alt1, Prodh, Hpd*, and *Otc* [101]. It was later identified that both gluconeogenesis and other aspects of amino acid metabolism and nitrogen turnover exhibit rhythmicity, which is controlled by Basic Helix-Loop-Helix ARNT Like 1 (Bmal1)-mediated rhythmic KLF15 expression [102]. Mice deficient in KLF15 expression abolished the rhythmic pattern in amino acid utilization and ammonia detoxification, highlighting the significance of KLF15 as an orchestrator of endogenous nitrogen balance in response to feeding/fasting cycles and day/night timings [102].

### 3.5. Bile Acid Synthesis and Metabolism

The liver is the primary organ for the synthesis and secretion of bile acids to the gall bladder. Bile acid synthesis is an important catabolic pathway for cholesterol and is important for preventing the accumulation of excess cholesterol and TG in the liver and for the absorption of dietary constituents from the intestine [103]. Moreover, the secreted bile acids act as important signaling molecules that act primarily via nuclear FXR or G-protein coupled bile acid receptor (Gpbar-1 or TGR-5) [104]. Cholic acid (CA) and Chenodeoxycholic acid (CDCA) are the primary bile salts (C24) in humans, synthesized from cholesterol through the concerted action of 17 distinct enzymes located in the cytosol, mitochondria, endoplasmic reticulum, and peroxisomes in hepatocytes [103]. There is a classical and an alternative pathway of bile acid synthesis. These are catalyzed by different enzymes [105]. The major rate-limiting enzyme for the classical pathway is cholesterol 7α-hydroxylase (CYP7A1) located in ER, whereas mitochondrial CYP450 enzyme sterol 27-hydroxylase (CYP27A1) initiates the alternative pathway [105]. Bile acids are stored in the gall bladder and get released into the intestine in response to cholecystokinin [103]. Subsequently, they are recirculated from the intestine back to the liver. Various enzymes and transporters in this pathway are regulated by FXR [106], which reduces the transcription of *CYP7A1*.

**Figure 3:**
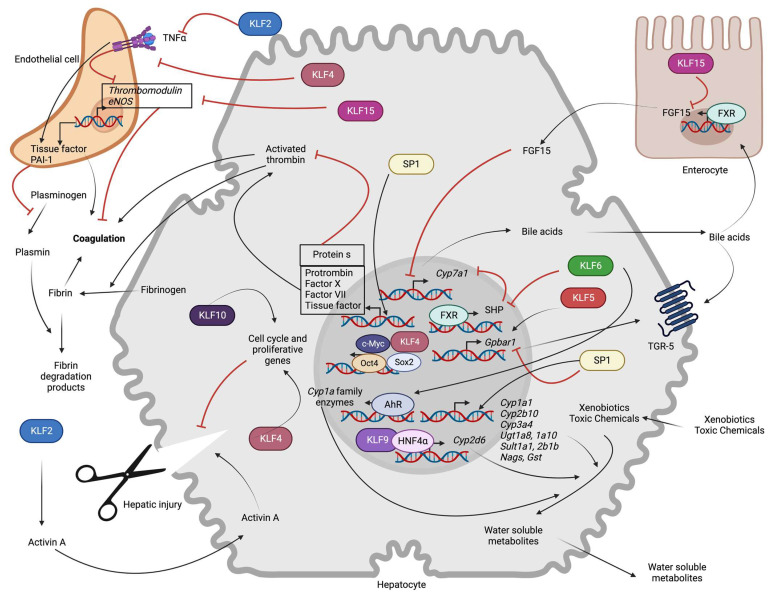
Role of hepatocyte SP/KLF transcription factors in non-metabolic functions: KLF6 indirectly increases expression of the rate-limiting enzyme for bile acid synthesis (Cyp7a1) by repressing FXR-mediated transcription of SHP. KLF15 indirectly promotes *Cyp7a1* transcription via transcriptional repression of FGF15 expression in enterocytes. KLF5 enhances transcription of the *Gpbar1*, whereas SP1 inhibits it. KLF6 promotes AhR-mediated transcription of the Cyp1a family, and KLF9 promotes HNF4α-mediated transcription of Cyp2d6. SP1 induces transcription of phase 1 (Cyp1a1, Cyp2b10, Cyp3a4) and phase 2 (Ugt1a8, Ugt1a10, Sult1a1, Sult2b1b, Nags, Gst) metabolic enzymes. SP1 also increases the transcription of clotting (Prothrombin, Factor X, Factor VII, TF) and anti-coagulation (Protein S) proteins. KLF2 and KLF4 prevent thrombosis and inflammation-induced prothrombotic gene (TF, PAI-1) expression and inhibitory influence on anti-thrombotic (TM, eNOS) gene expression. Endothelial KLF15 promotes thrombosis. In hepatocyte injury, endothelial KLF2 indirectly inhibits hepatic regeneration by leading to Activin A secretion. KLF4 and KLF10 promote hepatic regeneration by regulating the expression of cell cycle genes (AhR: Aryl hydrocarbon receptor, c-Myc: c-Myc oncoprotein, Cyp1a1: Cytochrome P450 Family 1 Subfamily A Member 1, Cyp2b10: Cytochrome P450, family 2, subfamily b, polypeptide 10, Cyp2d6: Cytochrome P450 family 2 subfamily D member 6, Cyp3a4: Cytochrome P450 family 3 subfamily A member 4, Cyp7a1: Cytochrome P450 family 7 subfamily A member 1, eNOS: Endothelial nitric oxide synthase, FGF15: Fibroblast growth factor 15, FXR: Farnesoid X receptor, Gpbar1: G protein-coupled bile acid receptor 1, Gst: Glutathione-s-transferase, HNF4α: Hepatocyte nuclear factor-4 alpha, KLFs: Krupple like family transcription factors, Nags: N-acetylglutamate synthase, Oct-4: Octamer-binding transcription factor 4, PAI-I: Plasminogen activator inhibitor 1, SHP: Small heterodimer partner, Sox2: SRY-Box transcription factor 2, SP: Specificity protein family transcription factors, Sult1a1: Sulfotransferase family 1A member 1, Sult2b1b: Sulfotransferase family cytosolic 2B member 1, TGR-5: Takeda G-protein receptor 5, TNF-α: Tumor necrosis factor α, Ugt1a8: UDP glucuronosyltransferase family 1 member A8, Ugt1a10: UDP glucuronosyltransferase family 1 member A10).

The role of SPs and KLFs in bile acid synthesis and signaling is relatively understudied and is summarized here in Figure 3. The primary evidence for the involvement of KLFs has been demonstrated in a study showing the involvement of KLF15 in the circadian control of bile acids. This study showed that CYP7A1 and CYP7B1 enzymes exhibit circadian patterns of expression, which are impaired in systemic *Klf15^−/−^* mice [107]. However, this regulatory mechanism does not occur in hepatocyte-specific *Klf15* knockout mice, indicating extrahepatic regulation of BA synthesis by KLF15 [107]. Later, they demonstrated that KLF15 suppresses *Fgf15* expression in enterocytes that negatively regulates BA synthesis. Gene reporter assays and ChIP assays revealed that the negative regulation of FGF15 by KLF15 is accompanied by KLF15 enrichment in the promoter region of *Fgf15* [107]. Accordingly, ileum obtained from *Klf15*^−/−^ mice showed significantly higher FGF15 transcription, confirming the role of the KLF15-FGF15 axis in bile acid synthesis and bile production [107]. Later, it was found that the DPP4 inhibitor, Teneligliptin, increases bile acid synthesis and prevents accumulation of cholesterol and TG in a high-fat diet-fed mouse by stimulating the expression of KLF15, which prevents FGF15 upregulation and bile acid synthesis [108]. 

Hepatocyte KLF6 has been implicated in the prevention of primary sclerosing cholangitis (PSC) [109]. Higher levels of liver KLF6 in PSC patients correlated with superior survival. Data from animal studies showed that hepatocyte-specific deletion of *Klf6* attenuated bile duct ligation-induced cholangitis, inflammation, and liver injury [109]. Further molecular studies in HepG2 cells associated the effect of KLF6 with transcriptional control of the *Nr0b2* gene by FXR, indicating possible crosstalk between KLF6 and FXR signaling pathways [109].

A role for SP1 and KLF5-mediated transcriptional regulation of TGR-5 has also been studied. Both transcriptional factors bind the promoter of TGR-5, with SP1 being a suppressor and KLF5 serving as an activator via either direct binding or indirectly via SP1 phosphorylation [110] that precludes binding of the latter.

In addition to these findings, a recent study also reported that KLF4 mediates TGR-5-induced intestinal metaplasia [111]. Molecular studies revealed that treatment of gastric epithelial cells with bile acids stimulates TGR-5 and activates HNF4α, which binds on *Klf4* and *Cdx2* promoters and stimulates metaplasia-related marker expression. This implicates KLF4 in extra-hepatic bile acid signaling [111].

### 3.6. Blood Coagulation

Hepatocytes produce major coagulation factors (except for factor VIII) and the components of the fibrin system [112]. The majority of the Vitamin K-dependent coagulation factors, such as Factor II, VII, IX, X and Protein C, Protein S, Factor V, XIII, fibrinogen, anti-thrombin, plasminogen, and plasminogen inhibitor are produced from liver parenchyma. Hence it is not surprising that liver damage can lead to a disturbance in the coagulation cascade, thus leading to hemostatic diseases [112]. 

SP family transcription factors have been found to regulate the transcription of almost all Vitamin K-dependent serine protease coagulation factors (Figure 3). Analysis of the human prothrombin (*F2*) promoter region revealed potential binding sites for HNF4, HNF3-β, and SP1 [113]. Electron mobility shift assays (EMSA) and site-directed mutagenesis in hepatocytes confirmed that SP1 regulates the expression of *F2* via direct promoter binding [113]. Factor X is another Vitamin K-dependent procoagulation factor that is produced in hepatocytes. A recent study identified that the promoter region of human Factor X contains two putative SP factor binding sites (−165 to −132 and −195 to −169) that can occupy both SP1 and SP3 [114] that have additive effects in the promoter activity [114]. Earlier mouse studies had identified potential binding sites for SP1 in the promoter region of Factor VII [115]. Similarly, another study showed that SP1 controls the transcription of the Factor VII gene [116]. Furthermore, analysis of blood samples obtained from a French-Canadian family patient with hemarthroses and chronic arthropathy identified a point mutation (C→G inversion) at −94 bp site in the 5’ flanking region of Factor VII that severely impaired its transcription [116]. Using in vitro reporter assays, they have identified that this site is responsible for the binding of SP1 and other nuclear factors [116]. A potential consensus binding site for SP family transcription factors has also been identified for tissue factor (*F3*, coagulation factor III) in endothelial cells and monocytes. Using DNase I footprint analysis and EMSA, authors in these studies have demonstrated constitutive binding of SP1 to the promoter region of *F3* and regulation of its expression along with NF-κB and AP-1 [117,118]. Although this has been exclusively studied in monocytes and aortic endothelial cells, the regulation of tissue factor expression by SP factors in the liver cannot be ruled out. 

The expression of pro-coagulation factors is not the sole way by which SP factors influence the coagulation cascade; they also regulate anti-coagulation mechanisms by controlling the expression of Vitamin K-dependent plasma glycoprotein, protein S. Multiple reports have characterized the binding sites for the SP1 and SP3 factors in the promoter of Protein S [119]. EMSA and ChIP assays in HepG2 cells identified at least four SP binding sites in the promoter region of Protein S, out of which the two sites close to the transcription start site (TSS) are indispensable for the constitutive expression of Protein S [119,120]. In another study, an SP1 binding site has been identified in the 5’ adjacent region of the Protein S promoter in both HepG2 and PLC (hepatoma) hepatic cells [120], underscoring the crucial significance of SP-mediated transcription in the coagulation cascade.

On the other hand, very few KLFs have been linked to thrombosis, and their role is mostly studied in endothelial cells (Figure 3). Studies in HUVEC cells suggested the possible involvement of KLF2 on the transcriptional induction of thrombomodulin (TM) and endothelial nitric oxide synthase (eNOS) with direct promoter binding [121]. Conversely, KLF2 reduced the expression of pro-thrombotic factors such as plasminogen activator inhibitor 1 (PAI-1) and cytokine-mediated induction of tissue factor (TF) [121]. Supporting these observations, KLF2 overexpression in endothelial cells increases the clotting time and flow rates, whereas knockdown produced the opposite effects, implicating KLF2 as a novel regulator of thrombotic endothelial function. Overall, these results suggest that KLF2 prevents the inflammatory cytokine-mediated inhibition of anti-coagulant TM expression and induction of procoagulant, PAI-1, and TF expression. Thus, KLF2 regulates vascular homeostasis under inflammatory conditions and is found to be important for the prevention of atherosclerosis and thrombotic events [122]. KLF2 also drives the expression of anti-thrombotic thrombomodulin (TM) in cooperation with ETS related gene (ERG), especially under high shear stress [123]. Despite the role of KLF2 in the regulation of TM expression and the anti-thrombotic effects, KLF2 deletion in mice does not result in spontaneous thrombosis. This indicates a possible functional overlap of KLF2 with other transcription factors [123]. Nevertheless, in addition to KLF2, KLF4 also plays an important vasoprotective role in preventing inflammation-induced thrombosis by increasing the expression of TM and eNOS and reducing the expression of VCAM-1 and TF [124].

In contrast to the vasoprotective effects of KLF2 and KLF4, KLF15 promotes thrombosis by inhibiting eNOS expression in a mouse model of deep vein thrombosis [125]. It remains to be established if hepatocyte KLF transcription factors also regulate the expression of coagulation factors.

### 3.7. Xenobiotic Metabolism

Chemicals ingested in the body pose a threat to different organ systems. While hydrophilic chemicals can be eliminated by the kidneys, the lipophilic molecules need to be converted into hydrophilic molecules before they can be excreted [126]. The process of converting a lipophilic molecule into a hydrophilic molecule is achieved in the liver [126]. The underlying metabolic pathways are broadly classified into Phase I and Phase II reactions. Phase I reactions involve oxidation, reduction, and hydrolysis by Cytochrome P450 (CYP450) family enzymes that can yield polar, water-soluble molecules [127]. Phase II reactions involve the conjugation of a hydrophilic moiety to the non-polar molecule to make them more soluble. Phase II reactions involve glucuronidation, glutathione conjugation, acetylation, sulfation, methylation, and amino acid conjugation, which are achieved by a separate set of hepatic enzymes for each reaction [127]. 

The cytochrome P450 superfamily of enzymes acts as oxygenases with heme as a cofactor. They are important for the biosynthesis of endogenous steroid hormones and the metabolism of fatty acids and exogenous xenobiotics [128]. Humans have around 57 different members of these enzymes classified into 18 different families [129]. Transcriptional profiling in hepatocytes identified the importance of HNF4α and other transcription factors for the expression of various P450 families of enzymes [130], including SPs that regulate Cyp1a1 [131], Cyp2b10 [132], and Cyp3a4 [133] (Figure 3). Cyp1a1 controls the biotransformation of many steroids and xenobiotics. Analysis of the porcine *Cyp1a1* gene promoter revealed SP1 binding site that was confirmed with EMSA and ChIP [131]. Similarly, functional mapping of the *Cyp2b10* promoter identified a possible binding site for SP1, whose occupancy is increased by PPARβ/δ in response to alcohol consumption resulting in increased *Cyp2b10* transcription [132]. However, it is not clear from this study if SP1 binding to Cyp2b10 promoter is also influenced under physiological conditions and to what extent SP1 controls physiological *Cyp2b10* transcription [132]. Studies in porcine primary hepatocytes also identified two cis-regulatory elements in the in the 5’ flanking region of *Cyp3a46* that bind SP1 and HNF1α, respectively, with and control its constitutive expression [134]. Later, it was identified that SP1-mediated regulation of Cyp3a extends to the other members of this family and operates similarly in both porcine and human hepatocytes. It was found that both transcription Factor Y and SP1 cooperate in regulating the expression of six members of the Cyp3a family [135]. 

The evidence for the existence of KLF binding sites in the promoters of Cyp enzymes is relatively sparse. Cyp2d6 is a major hepatic enzyme responsible for the metabolism of many marketed drugs. Deletions and mutations in the promoter region of *Cyp2d6* identified a KLF9 binding site (−22/−14 region) [136]. However, it was found that KLF9 itself is a weak inducer of *Cyp2d6* expression; its inducing effect relies mostly on HNF4α transactivation [136]. In another study, it was accidentally observed that nematode KLF1 increases lifespan in mitochondrial gene mutants through transcriptional induction of xenobiotic metabolizing enzymes [137]. Particularly, KLF1 pull-down, and ChIP sequencing identified binding sites of KLF1 in the promoter regions of four CYP450 oxidases, including *Cyp13a8, Cyp37a1, Cyp13a11,* and *Cyp33c9* [137]. It needs to be determined if KLF1 has a similar role to play in the induction of mammalian hepatic CYP450 oxidases. KLF6 was identified to regulate aryl hydrocarbon receptor (AhR) expression, which in turn controls the majority of Cyp enzymes and phase-ii metabolizing enzyme expression by binding to xenobiotic response element (XRE) [138].

Phase II metabolic reactions are catalyzed by a separate set of enzymes, which include UDP-glucuronosyltransferases, N-acetyl transferases, methyl transferases, sulfotransferases, amidases, glutathione-s-transferases and several amino acid transferases [127]. Synthesis and expression of these enzymes depend on diverse transcription factors, which include NF-E2 related factor-2, C/EBPβ, hepatic nuclear factor 1 (HNF1), PPARs, pregnane x receptor (PXR), and FXR. Two SP1 binding sites (a transcription site and an upstream site in the TATA box) have been identified in the 5’ flanking region of the UDP-glucuronosyltransferase enzymes *UGT1A8*, *1A9*, and *1A10* in colon cells [139]. These enzymes are predominantly expressed in extrahepatic tissues (colon), and SP1 binding to the upstream site greatly enhanced the promoter activity of UGT1A8 and UGT1A10 enzymes [139]. Similarly, SP1 was also found to regulate the transcription of human sulfotransferases *SULT1A1* [140] and *SULT2B1b* [141], N-acetyl glutamate synthase (NAGS) [100], and pi class glutathione-s-transferase [142] by direct binding in the promoter region of these enzymes.

Overall, from the existing evidence, it is clearly evident that SP1 controls the transcriptional induction of several metabolic enzymes (Cyp1a1, Cyp2b10, Cyp3a family, UGT1A8-10, SULTA1, SULT2B1b, NAGS, pi class GST) by direct binding to the promoter region of these genes under physiological conditions as well in response to the drug exposure (Figure 3). The role played by KLF factors in the expression of these hepatic enzymes is relatively understudied. Although some genes have noticeable KLF binding sites, their potential to promote the expression of these enzymes on their own is still questionable, and they may act in cooperation with other transcription factors to influence their transcription.

### 3.8. Liver Regeneration

The liver is one of the organs that have remarkable regeneration capacity following injury [143]. Animal models of partial hepatectomy and drug-induced liver injury have been of immense help in deciphering the mechanisms of hepatic regeneration. Hepatic regeneration involves signaling cascades associated with growth factors, cytokines, matrix remodeling, and feedback regulation of growth-related signals [144]. 

Primary murine hepatocytes grown in collagen monolayers display a high proliferation rate, which is associated with increased mitogen-activated protein kinase (MAPK) signaling and increased binding of ETF transcription factor (ETF), E2F family of transcription factor (E2F) and SP1 [145]. The same signaling events also occur in mouse liver after partial hepatectomy [145]. KLF transcription factors also influence hepatic regeneration. For instance, KLF2, which is predominantly expressed in endothelial cells, negatively regulates regeneration via the secretion of activin A [146]. Supporting this observation, endothelial cell-specific loss of KLF2 augmented hepatocyte proliferation and reduced CCL4-induced liver damage [146]. In contrast to the role of KLF2, KLF4 is pro-regenerative both in in vitro models of hepatocyte differentiation from embryonic stem cells and in vivo. In the earlier studies, iPSCs were successfully differentiated into hepatic cells with four programming factors that include KLF4 (Oct-4/Sox2/Klf-4/c-Myc) [147]. Another study showed that induction of KLF4 expression via plasmid injection in mice or adenovirus-mediated overexpression in cultured LX-2 cells attenuated CCL4-induced injury or TNF-α-mediated cell damage in vitro by inhibiting Apelin expression and signaling [148]. Similarly, *Klf10* knockout mice showed compromised liver regeneration following partial hepatectomy in mice [149] associated with higher expression of Smad3, p15, TGF-β1 and reduced expression of proliferative markers (cMyc and cyclin D1) [149]. So, these studies summarize that KLF4 and KLF10 may have pro-hepatic regenerative capabilities. KLF2 acts in the opposite way and suppresses hepatic regeneration (Figure 3).

## 4. SPs and KLFs in the Pathogenesis of Hepatic Diseases

**Figure 4:**
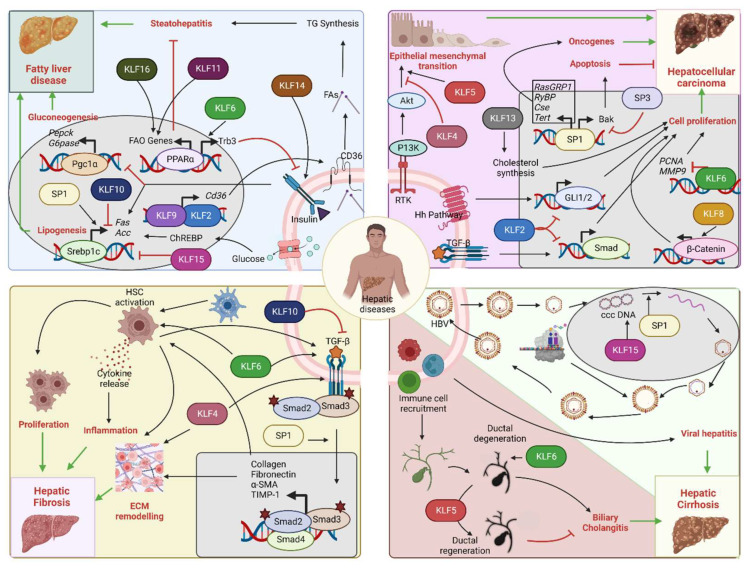
Role of SP/KLF transcription factors in hepatic diseases: Upper left corner: Insulin resistance/NAFLD—Insulin signaling promotes SREBF-1-mediated lipogenesis and inhibits PGC-1α-mediated gluconeogenesis. Impaired insulin signaling leads to fatty liver disease. KLF2 and KLF9 drive *Cd36* expression that increases fatty acid uptake and steatohepatitis. KLF11 and KLF16 drive PPARα-mediated fatty acid oxidation gene expression and prevent steatohepatitis. KLF6 interferes with insulin signaling through PPARα-mediated Trb3 expression. KLF14 promotes insulin signaling through PI3K-Akt. SP1 increases lipogenesis, while KLF10 and KLF15 inhibit it. Upper right corner: HCC—SP1 promotes transcription of oncogenes, such as RasGRP1, RYBP, CSE, and TERT, and facilitates HCC progression. SP1 can also increase the expression of the apoptotic gene Bak, hence promoting tumor suppression. SP3 opposes SP1 action on Bak expression. KLF2 is a tumor suppressor by inhibiting Hedgehog and TGF-β-Smad pathways. KLF8 and KLF13 promote tumor proliferation by activation of Wnt/β-catenin and cholesterol synthesis. KLF6 also acts as a tumor suppressor by inhibiting PCNA and MMP9 oncogene transcription. KLF5 facilitates receptor tyrosine kinase-mediated EMT, whereas KLF4 opposes it. Bottom left corner: Hepatic fibrosis—HSC activation promotes hepatic fibrosis by altering the ECM composition and releasing inflammatory cytokines. SP1 promotes fibrosis through stimulation of the TGF-β pathway. KLF4 and KLF6 promote fibrosis by facilitating the HSC actions on ECM remodeling and the TGF-β pathway. KLF10 opposes the actions of the TGF-β pathway and hence prevents the progression of fibrosis. Bottom right corner: Viral hepatitis and biliary cholangitis: HBV infection, ccc DNA replication, and coat protein synthesis lead to the formation of virion particles that are finally released. SP1 facilitates the replication of ccc DNA. KLF15 activates the promoter of surface proteins and core genes. Immune cell-mediated damage of biliary epithelial cells causes bile duct degeneration and cholangitis. KLF6 facilitates the development of cholangitis through excess bile acid secretion and accumulation, while KLF5 prevents it by promoting biliary epithelial cell remodeling and ductal regeneration (Acc: Acetyl coA carboxylase, Akt: Akt serine/threonine kinase, Bak: Bcl-2 homologous antagonist/killer, ccc DNA: Covalently closed circular DNA, CD36: Fatty acid translocase, ChREBP: Carbohydrate response element binding protein, Cse: Cystathionine γ-lyase, ECM: extracellular matrix, FA: Fatty acids, FAO: Fatty acid oxidation, Fas: Fatty acid synthase, GLI1/2: Glioma-associated oncogene 1/2, G6Pase: Glucose 6 phosphatase, HBV: Hepatitis B virus, Hh: Hedgehog, HSC: Hepatic stellate cell, KLFs: Krupple like family transcription factors, MMP9: Matrix metalloproteinase 9, PCNA: Proliferating cell nuclear antigen, Pepck: Phosphoenol pyruvate carboxykinase, Pgc-1α: Peroxisome proliferator-activated receptor-gamma coactivator-1alpha, PI3K: Phosphoinositide 3-kinases, PPARα: Peroxisome proliferator-activated receptor alpha, RasGRP1: RAS guanyl-releasing protein 1, Rtk: Receptor tyrosine kinase, RYBP: RING1 And YY1 binding protein, Smad: Suppressor of mothers against decapentaplegic, SP: Specificity protein family transcription factors, Tert: Telomerase reverse transcriptase, TG: Triglycerides, TGF-β: Transforming growth factorβ, Trb3: Tribbles pseudokinase 3).

As outlined in the previous sections, KLF and SP family members facilitate different physiological functions of the liver by regulating gene transcription in various cell types that are present in the organ. Several studies that have been conducted using animal and cell culture models that model liver diseases have improved understanding of the contribution of these transcription factors in different pathological conditions (Table 1). Importantly, DIO models enhanced knowledge about KLFs and their contribution to insulin resistance and NASH development. Moreover, the advent of novel molecular biology techniques and in vivo gene modulation by adeno-associated virus-mediated knockdown/knock-in approaches helped to appreciate their translational impact in several hepatic diseases. The following sections provide a detailed overview of the involvement of SP/KLF transcription factors in different hepatic diseases. For a simple overview, readers are encouraged to go through Figure 4 and Table 1.

### 4.1. Metabolic Diseases (Diabetes, Hyperlipidemia, and Hypercholesteremia)

Insulin exerts multiple hepatic effects that help control blood glucose levels. These include increased glucose uptake by liver cells and promotion of hepatic glycogenesis, fatty acid synthesis, and inhibition of hepatic gluconeogenesis [181]. Some of these effects are at least partially mediated through SP/KLF transcription factors. For instance, constitutive activation of hepatocyte FOXO1 inhibits both basal and insulin-stimulated hepatic lipogenesis by reducing the transcriptional activity of SP1 and SREBP1c and preventing the transcriptional initiation complex assembly at the promoter of SREBP1c [150]. It is well known that SREBP1c promotes hepatic lipogenesis via the regulation of fatty acid synthase (*Fas)* and acetyl-CoA carboxylase (*Acc)* promoters along with various transcriptional factors, including SP1 [182]. In addition, other studies established the role of insulin in hepatic SP1 transcriptional activity and its post-transcriptional regulation. Treatment of H-411E liver cells with insulin increased O-GlcNAcylation and phosphorylation of SP1 and altered its nuclear localization and expression of its target genes, such as Calmodulin [183]. Later, it was shown that treatment of hepatic cells with insulin also increased the nuclear content of SP3 in a PI3K-specific manner. It remains to be determined if these events have any relevance to hepatic insulin resistance. 

In a study that investigated with DNA affinity purification followed by LC/MS analysis, transcription factors that mediate the effects of insulin in hepatic glucose production, identified significant enrichment of the hepatocyte phosphoenolpyruvate carboxykinase (*Pck1*) promoter with KLF3 [184]. However, the study failed to describe the functional significance of this finding [184]. Studies in human populations identified an association of gene polymorphism in the *Klf6* gene, *KLF6-IVS1–27A,* that attenuates *Klf6* splicing with hepatic insulin resistance and delays NAFLD progression [54]. Further molecular characterization attributed this effect of KLF6 to increased hepatic *Gck* expression [54]. 

Previous studies have identified possible binding sites for KLF9, KLF10, and KLF14 in the promoter of *Ppargc1a* [60]. KLF10 and KLF14, which are upregulated in response to high-fat diet, promoted PGC-1α-induced hepatic gluconeogenesis, supporting their possible role in insulin resistance. Nevertheless, insulin signaling inhibits PGC-1α, while its overexpression promotes hepatic insulin resistance [185,186].

Similarly, KLF9 is induced in response to dexamethasone and promotes PGC-1α-mediated gluconeogenesis, indicating its potential involvement in glucocorticoid-induced diabetes [55]. In addition to these pre-clinical findings, gene polymorphism in the human *KLF14* gene has been associated with type-2 diabetes incidence [177]. Similarly, animal studies in hepatocyte-specific *Klf16* knockout mice showed increased plasma glucose levels associated with reduced insulin sensitivity and increased hepatic gluconeogenesis [84]. Impairment in hepatic insulin signaling in these mice corroborated with reduced phosphorylation of Akt and GSK-3β [84]. 

In addition, a possible role for hepatic KLF15 in ER stress-related insulin resistance has been identified in animal models [85]. Specifically, mice deficient in *Klf15* expression showed increased hepatic ER stress and inflammation. However, these mice are resistant to HFD-induced insulin resistance and NAFLD progression. Further molecular studies in these mice identified impaired mTOR activity in these mice, which may be the reason for uncoupling ER stress and insulin resistance [85].

The conclusive evidence from the literature thus supports a possible role for SP1 in insulin-stimulated hepatic lipogenesis and steatohepatitis, although it is not clear if genetic modulation of SP1 has any influence on hepatic insulin sensitivity and lipid accumulation.

### 4.2. Non-Alcoholic Fatty Liver Disease (NAFLD)

NAFLD describes the deposition of fat in the liver in people who ingest little or no alcohol. The causes for the development of NAFLD include underlying metabolic abnormalities such as obesity, type II diabetes, and high cholesterol [187]. If left untreated, such a condition can lead to severe cirrhosis and fibrosis, eventually resulting in hepatic failure. There are no treatments for NAFLD, other than treating the underlying cause [188]. 

Our knowledge of the possible role of SP/KLF family transcription factors comes from the animal studies employing DIO models and CCL4/western diet models that cause steatohepatitis. Very limited clinical data are available regarding the importance of these transcription factors to the human NAFLD. The ingenuity pathway analysis (IPA) of publicly available human datasets from patients with NAFLD/NASH consistently predicted the possible association of SP1 with NASH incidence (GSE89632) and fibrotic disease (GSE130970) and KLF2 association with a particular cohort with advanced fibrosis (GSE130970) [160]. An indirect role has been proposed for SP1 in the epigenetic regulation of Paraoxonase 1 (PON1), a detoxification enzyme secreted by the liver and known to metabolize LDL and HDL particles. These authors have shown that genetic polymorphism in the gene regulatory region of PON1 (rs705379:C  >  T) contributes to NAFLD pathogenesis by increasing the SP1-mediated promoter occupancy leading to hypomethylation and PON1 expression and simultaneous SREBP-2 upregulation-mediated cholesterol accumulation in the liver [151].

With a system biology approach applied to human liver transcriptomic datasets to identify the gene co-regulatory networks and transcriptional regulation in a disease state, it was identified that KLF13 is one of the most significantly enriched transcription factors associated with NAFLD disease pathogenesis along with SREBF2, HNF4A, SREBF1, and YY1. Further, increased expression of KLF13 is validated in NAFLD patient liver samples compared with the control group. Moreover, network analysis revealed LDLR gene is the most highly regulated gene by these transcription factors [23]. 

### 4.3. Hepatocellular Carcinoma (HCC)

HCC represents the most common primary form of cancer in the liver [189]. It develops as a secondary event to viral hepatitis or non-alcoholic liver disease [189]. An altered microenvironment in the presence of viral infection or chronic inflammation compromises the genomic integrity resulting in mutations in the genome that can ultimately lead to HCC progression [189]. 

SP transcription factors have been identified that regulate the transcription of various oncogenes that have been linked to HCC. These include Ras guanine nucleotide-releasing protein 1 (RasGRP1), RING1 and YY1 binding protein (RYBP), and cystathionine γ-lyase (CSE) [152]. The regulatory elements of these genes bind SP factors that increase their expression, which in turn facilitates tumor growth and metastasis [152]. SP transcription factor binding sites are also present in the promoter region of telomerase reverse transcriptase (TERT) in HCC cells. SPs bind on these sites along with nuclear receptor coactivator-3 (NCOA3) and transactivate *TERT* expression [153]. In contrast to the activation of oncogene expression, SP1 also stimulates the expression of the pro-apoptotic *Bak* gene along with ZBP-89 [156]. Surprisingly, SP3 has the opposite influence on *Bak* gene expression by binding to the SP1 site and preventing SP1-mediated Bak expression [156]. Furthermore, SP transcription factors increase the expression of various long non-coding RNAs, which promote liver cancer progression [154]. Using RNA interference studies in HepG2, SNU-449, and SK-Hep-1 cells, it has been shown that SP1, along with SP3 and SP4, regulate various long non-coding RNAs that are highly upregulated in liver cancer (HULC) [154]. Further, metformin and other anti-neoplastic agents reduce the expression of both SP transcription factors and the HULC lncRNAs, thus holding therapeutic potential for HCC management [154]. Interestingly, metastasis-associated lung adenocarcinoma transcript 1 (MALAT1) is a lncRNA that is highly expressed in metastatic lung cancer. Co-silencing of both SP1 and SP3 or treatment with Mithramycin A (SP transcription inhibitor) repressed MALAT1 expression in HCC cells [155], which suggests another potential pathway via which SP transcription factors may contribute to HCC. 

KLFs have multiple effects on HCC progression. KLF6 is one of the most characterized transcription factors for its involvement in HCC. In an earlier study, it was shown that KLF6 expression is lost in cancerous tissues obtained from HCC patients and HCC cell lines (HepG2 and Hep3B), suggesting that it may act as a tumor suppressor in liver carcinogenesis [190]. This observation was further strengthened by the findings that observed mutations in the *KLF6* gene in cancer tissue specimens obtained from HCC patients. Accordingly, overexpression of wild-type *KLF6* but not mutant *KLF6* impaired G1 to S transition in cultured HepG2 cells [191]. In a more recent study, these findings were recapitulated in vitro and in vivo. Overexpression of *KLF6* reduced cell proliferation and decreased cell invasive potential by reducing the expression of *PCNA* and *MMP-9* in HCC cells, eventually leading to suppression of tumor growth in vivo [169]. In contrast to these findings, RNA interference against *KLF6* in HepG2 and HuH7 cells caused upregulation of pro-apoptotic p53 and inhibited anti-apoptotic Bcl-xL expression, implicating the possible importance of KLF6 for HCC cells to evade apoptosis [170]. 

KLF13 expression is increased in HCC tissues analyzed from The Cancer Genome Atlas (TCGA) database [176]. Gene silencing and overexpression studies in HCC cell lines clearly supported the oncogenic role of KLF13, which was later proven to be through enhanced cholesterol synthesis in a HCC xenograft mouse model [176]. KLF5 is also appreciated for its tumor-promoting role in HCC through the facilitation of epithelial-mesenchymal transition pathways such as PI3K/Akt/Snail signaling [167]. In striking contrast to this observation, overexpression of KLF4 in HCC cells prevented EMT transition by reducing the expression of Slug [163], highlighting the quite differential roles of KLF transcription factors in hepatic carcinogenesis. KLF4 has also been studied as a prognostic marker in HCC patients after curative resection. Overall survival rate and lack of recurrence in patients undergoing hepatectomy were reduced in patients with lower KLF4 expression rather than in patients with higher KLF4 expression [192]. 

KLF2 is mostly observed to have tumor-suppressing effects in HCC cells as it inhibits cell proliferation via suppression of Hedgehog signaling and metastasis via prevention of TGF-β-mediated Smad signaling [161]. However, confusion was created by a study showing that KLF2 expression is increased in HCC and promotes cell proliferation via direct binding to c-myc [162]. Recent studies have indicated the pro-tumorigenic potential of KLF8 in HCC progression through activation of the Wnt/β-catenin signaling pathway [174]. 

Overall, from the enlisted evidence, the pro-oncogenic role has been clearly established for KLF5, KLF13, and KLF8. Whereas for the SP1, KLF6, and KLF2, discordant findings have been reported as to whether they play a tumor-promoting or suppressing role in HCC progression. More studies need to be done to establish a clear disease association for these transcription factors and for the other family members that have not been studied yet.

### 4.4. Hepatic Fibrosis and Cirrhosis

Liver fibrosis occurs with excessive accumulation of connective tissue and secretion of matrix components by activated fibroblasts that replace healthy hepatic parenchyma with non-functional fibrotic scar [193]. Fibrosis results in liver failure that necessitates hepatic transplantation in chronic stages. Various pathological stimuli, including viral infection, excessive alcohol intake, or metabolic dysfunction as occurs in type II diabetes and non-alcoholic fatty liver disease, can induce fibrosis [193]. Apart from fibroblasts, hepatic stellate cells and immune cells have important roles in the progression of fibrosis. Fibrogenic cytokines such as TGF-β, Angiotensin-II, and leptin activate these cells and lead to fibrotic remodeling [194,195].

The contribution of SP/KLF transcription factors for their role in the activation of fibrotic genes has been studied in several animal models. The pro-fibrotic effects of SP1 were revealed in a study where SP1 decoy, double-stranded ODN transfection in hepatic stellate cells (HSC-T6) prevented fibrosis-related gene expression by preventing binding of SP1 to the promoter of TGF-β1, Platelet-derived growth factor (PDGF)-β and α-SMA [157]. Another study compared existing microarray datasets of hepatic RNA obtained from humans and revealed higher expression of SP1 in patients with hepatic fibrosis [196]. 

Although various KLFs have been associated with hepatic steatosis and fatty liver disease (described in Section 3.3), only a few KLFs have been studied for their direct involvement in fibrotic signaling. Along with SP1, KLF6 regulates the transcription of the α1 collagen gene and activates hepatic stellate cells [171]. In addition to the α1 collagen, fibrogenic TGF-β activation, expression of TGF-β receptors, and urokinase plasminogen activator are controlled by KLF6, which occupies GC-rich promoter regions [171]. Similarly, KLF4 is pro-fibrotic, as shown with reduced hepatic fibrosis following treatment with siRNA against KLF4 [164]. This study revealed that KLF4 promotes fibrosis in hepatic stellate cells by augmenting TGF-β signaling and disturbing MMP-1 vs. TIMP-1 balance that alters extracellular remodeling [164]. Contrary to these findings, anti-fibrotic effects of KLF10 were established in an animal model of high sugar intake, as livers of *Klf10* KO mice displayed higher fibrosis and collagen accumulation following HSD [79]. 

The conclusive evidence from these studies clearly highlighted that certain members of the SP/KLF family (SP1, KLF6, and KLF4) promote the transcriptional regulation of fibrotic remodeling, facilitating the incidence and progression of liver fibrosis. Among these, SP1 targeting has proved beneficial against fibrosis in an animal model of liver fibrosis. It needs to be determined if these therapies can make it into the clinical stage. 

### 4.5. Viral Hepatitis

Acute viral hepatitis is caused by infection with Hepatitis A, B, C, D, or E viruses that induce hepatic inflammation [197]. Less commonly, hepatitis can also be caused by Cytomegalovirus, Epstein Barr virus, Herpes simplex virus, and yellow fever virus. The immune system is activated to contain the viral infection from hepatocytes and clear the infected cells. The immune response to viral infection occurs at the cost of self-damage to liver tissue leading to hepatic inflammation that can gradually develop into hepatic fibrosis and liver failure [198].

SP and KLF transcription factors have multiple roles in the pathogenesis of viral hepatitis, especially with the hepatitis B virus (HBV). The HBV genome contains multiple SP1 binding sites [199]. Multiple other transcription factors seem to exert synergistic effects with SP1 in coordinating the replication of HBV covalently closed circular (ccc) DNA [158]. In fact, SP1-mediated HBV replication can be targeted with drugs that can treat hepatitis B. Screening for alpha-glucosidase (AG) inhibitors against the HBV infection in hepatocytes identified one candidate inhibitor that lowered ccc DNA production through binding to SP1 [200]. Similarly, HBV genome expression is suppressed in human hepatoma HuH7-cells that are subjected to TNFα treatment or p65 overexpression, which seems to suppress SP1-mediated transcriptional activity [201]. 

Among the KLFs, KLF15 is well-studied for its influence on HBV replication. KLF15 binds to the HBV genome and activates promoters of surface and core genes [179].

### 4.6. Biliary Cholangitis

Primary biliary cholangitis is an autoinflammatory disease-causing destruction of bile ducts [202]. Progressive cholangitis can cause hepatic damage, fibrosis, and cirrhosis [202]. Biliary epithelial cells undergo ductular regeneration following liver injury. Defects in this dynamic remodeling process also account for biliary cirrhosis. KLF5 controls this crucial process in biliary epithelial cells, especially under cholestatic injury conditions [168]. Absence of KLF5 from all hepatic epithelial cells but not exclusively in hepatocytes compromises biliary epithelial cell remodeling, aggravates cholestatic injury, and increases mortality upon 3,5-diethoxycarbonyl-1,4-dihydrocollidine (DDC) administration [168]. Hepatic RNA-seq analysis of biliary epithelial cells isolated from liver-specific *Klf5* KO (KLF5 absence in hepatocytes and biliary epithelial cells) mice upon DDC treatment showed reduced expression of cell cycle genes, highlighting the importance of KLF5 for the biliary ductal regeneration [168].

### 4.7. Hemochromatosis

In hereditary hemochromatosis, the body absorbs too much iron from food leading to excessive iron deposition in the liver and other internal organs [203]. Mutations in the hemochromatosis gene (HFE) gene, which encodes for a cell surface receptor that is co-expressed with β2 microglobulin, is causal for hereditary hemochromatosis [203]. Analysis of the human HFE gene has revealed multiple SP1 binding sites in the promoter region. Functional studies revealed the transactivation of the HFE gene by SP1, along with other transcription factors such as C/EBPalpha and GATA-1 [204]. Further studies also revealed that SP1 enhances HFE transcription indirectly by preventing poly ADP ribose polymerase 1 (PARP1) association with an HFE promoter, thereby interfering with iron-mediated feedback regulation of the HFE gene [205]. However, it remains to be discovered if human hereditary hemochromatosis is associated with impaired SP1-mediated transcription of the HFE gene.

### 4.8. Wilson’s Disease

Wilson’s disease (WD) is an autosomal recessive disease characterized by the accumulation of copper in the liver due to mutations in a gene that encodes copper-transporting P-type ATPase (ATP7B), which mediated copper efflux from the cells [206]. It can lead to steatosis, cirrhosis, and hepatic failure [206]. Excessive hepatic copper levels have been also shown to reduce the transcriptional activity of the hepatic nuclear receptors, FXR, RXR, HNF4α, and LRH-1 in *Atp7b*^−/−^ mice and patients with WD [207]. As with iron metabolism, SP1 regulates copper metabolism in mammalian cells by stimulating the human copper transporter (*hCtr1*) that facilitates copper entry into the cells [208]. Luciferase reporter assays identified 3 possible SP1 binding sites in the promoter of the *hCtr1* gene [208]. Hence, additional studies investigating a potential direct causal relationship of SP/KLF transcription factors in the pathophysiology of WD are warranted.

### 4.9. Drug-Induced Hepatic Toxicity

Drug-induced liver injury is one of the most common reasons for drug withdrawal from the market [209]. Despite the role in the expression of several drug-metabolizing enzymes, SP1 was not identified as a responsive transcription factor in a cell-based reporter assay testing 64 hepatotoxic compounds [210]. On the other hand, KLF6 is involved in diclofenac-induced hepatic injury in a canine model [172]. Specifically, chronic oral administration of diclofenac for 28 days in beagle dogs, caused severe liver injury characterized by steatosis, glycogen depletion, apoptosis, acute lobular hepatitis, and interstitial inflammatory cell accumulation. Further, immunohistochemical findings revealed, increased KLF6 nuclear expression in Kupffer cells and migrating monocytes that sustain hepatic inflammation [172]. 

### 4.10. Acute Hepatic Failure

Acute liver failure is a life-threatening condition due to sudden—within days to weeks—loss of hepatic function characterized by jaundice, coagulopathy, elevated hepatic enzyme levels in the blood and hepatic encephalopathy [211]. The most common causes of acute liver failure include viral hepatitis and exposure to high doses of hepatotoxic compounds, such as acetaminophen, alcohol, or CCL4 [211]. KLFs have been well studied for their role in acute liver injury. Particularly, hepatic KLF6 is elevated in patients with acute liver failure and in mice subjected to CCL4 or acetaminophen administration [173]. Further, this study showed that KLF6 is a direct transcriptional inducer of autophagy-related genes, such as *Atg7* and *Becn1,* in a p53-dependent manner, implicating autophagy in KLF6-dependent cell death in acute liver injury [173]. Unlike KLF6, KLF4 and KLF15 reduce the severity of acute liver injury and promote liver regeneration. For instance, KLF15 attenuates endotoxemia-induced hepatic injury by suppressing p38 MAPK/ERK1/2 signaling, apoptosis, and inflammation in hepatocytes and Kupffer cells [180]. KLF4 is part of the 3-gene cocktail (Oct4/Sox2/Klf4), which is used to differentiate iPSCs from hepatocyte-like cells [165]. In a mouse model of CCL4-induced acute liver injury, administration of a 3-gene cocktail or iPSC-Heps attenuated hepatic necrosis and oxidative stress and improved hepatic function and survival [166]. Another study indicated a key role for SP1 in the progression of CCL4-induced hepatic fibrosis through the transcriptional promotion of TGF-β. Intravenous administration of a decoy oligonucleotide against SP1 (R-SP1 decoy ODN), effectively suppressed hepatic fibrosis in these mice by inhibition of TGF-β signaling, and suppression of inflammatory cell accumulation and macrophage activation [159]. Hence from these studies, it is understood that SP1 and KLF6 have a detrimental role to play in acute hepatic injury, KLF4 and KLF15 act in an opposite way to promote hepatic regeneration and thereby attenuate the acute injury induced by several toxic chemicals.

## 5. Epilogue

In the early years of hepatic transcriptional regulation research, it was believed that four protein families (C/EBP, DBP, HNF3, and HNF4 families) were the main regulators of genes that are important for liver biology [212]. Later, through the advent of modern biological techniques, the contribution of other transcription factors, including SP/KLF transcriptional factor family members, was revealed [213]. The contribution of these transcription factors for the gene transcription may be context-dependent, i.e., gene transcription is very complex and widely influenced by the environmental factors such as availability of food (fast/fed state), circulating hormones (Insulin, glucagon, glucocorticoids) and subjected to circadian regulation. Hence care should be exercised by the readers before interpreting the involvement of SP/KLF members in particular processes. However, the structural framework provided in this review, along with the figures, lays out a simpler and broad overview of these transcription factors and their influence on hepatic functions under physiological and pathological contexts.

Among various metabolic pathways, hepatic glucose production (gluconeogenesis), lipid uptake, de novo lipogenesis, β-oxidation of lipids, and cholesterol metabolism are the key processes that are regulated by KLF/SP family members (Figure 2). KLF9, KLF10, KLF14, and KLF15 stimulate hepatic gluconeogenesis in response to fasting and glucocorticoids [60]. These transcription factors influence the expression of two important enzymes controlling gluconeogenesis, *Pepck,* and *G6p,* either by direct binding to their promoter or by facilitating the PGC-1α-mediated transcriptional expression [60]. Interestingly, KLF11 does oppose these actions by inhibiting hepatic gluconeogenesis via transcriptional repression of *Pepck* [61]. SP1 and KLF6 increase *Gck* expression, making them possible candidates for control of hepatic insulin sensitivity [54,68].

Animal models of diet-induced obesity and genetic models of type-2 diabetes elucidated the contribution of SP/KLF members to hepatic fatty acid metabolism. KLF2 and KLF9 increase the transcriptional expression of fatty acid translocase, *Cd36,* and increase fatty acid uptake by hepatocytes [72,77]. KLF10 also influences CD36-mediated lipid uptake by hepatocytes via palmitoylation of CD36 that promotes translocation to the membrane [80]. KLF10 and KLF15 inhibit hepatic lipogenesis through distinct mechanisms. KLF11 increases hepatic fatty acid oxidation by facilitating the PPARα-mediated expression of enzymes controlling β-oxidation [81]. Whereas KLFs were mostly studied for glucose and fatty acid metabolism, SP1 has been identified to control the expression of several genes important for lipoprotein and cholesterol metabolism [89,90,91,92]. 

SP/KLF members also contribute to the non-metabolic functions of hepatocytes (Figure 3). KLF6 enhances the expression of the rate-limiting enzyme for bile acid synthesis, *Cyp7a1,* indirectly through inhibition of FXR and SHP [109]. In addition, KLF15 indirectly promotes *Cyp7a1* transcription by suppressing FGF15 expression in enterocytes [107]. Similarly, for clotting factor production, SP1 increases the transcription of several clotting factors [113,118]. On the other hand, KLFs were studied for their role in blood coagulation with respect to vascular endothelial cells. KLF2 and KLF4 were shown to be vasoprotective against inflammation-induced coagulation cascade [121,122,124]. For liver-specific metabolic enzyme expression, the ubiquitous transcription factor SP1 has been shown to regulate several phase I and phase II enzymes [131,132,133,134,140,141]. Similarly, for the regenerative capacity of hepatocytes, KLF4 and KLF10 were identified to be pro-regenerative [148,149], whereas KLF2 exerts opposite actions by increasing the Activin A signaling [146]. 

The importance of SP/KLFs for hepatic health is signified by their involvement, when they are dysregulated, in hepatic diseases, such as metabolic diseases, hepatocellular carcinoma, hepatic cirrhosis, etc. (Figure 4). The expression of KLF2 [72], KLF9 [77], and KLF14 [59] is increased in response to HFD in mice, and their inhibition attenuates steatohepatitis, indicating their involvement in NAFLD pathogenesis. Similarly, KLF6 increases PPARα activity post-transcriptionally and contributes to hepatic insulin resistance [76]. KLF11 and KLF10 are mostly protective against diet-induced obesity and fibrosis [79,81]. For other members of the SP/KLF family, such as KLF15, the role is more complex as there are reports that both support and oppose its contribution to hepatic insulin resistance and hypertriglyceridemia [85,86]. 

For HCC development, SP1 [152] and KLF8 [174] may have pro-tumorigenic potential as they regulate the expression of several oncogenes. KLF5 and KLF4 control the epithelial-mesenchymal transition, which is critical for the invasiveness and metastasis of HCC [163,167]. On the other hand, KLF2 and KLF6 have anti-proliferative activities as they oppose the hedgehog pathway [161] and PCNA, MMP-9 expression [169], respectively. For hepatic fibrosis, activation of HSC is a key event that controls different stages of fibrotic remodeling. KLF6 has been shown to be induced in response to HSC activation, but this seems to be a protective response as it suppresses target gene transcription and facilitates apoptosis [171]. Similarly, KLF4 and SP1 seem to promote TGF-β-mediated fibrotic signaling [157,164]. Conversely, KLF10 suppresses hepatic fibrosis by negating TGF-β signaling [79]. Regarding viral hepatitis, SP1 and KLF15 are important for the replication of the HBV genome [179,199]. On a different note, KLF5 seems to regulate the ductal regeneration ability of biliary ducts after the injury and thus may attenuate biliary cholangitis [168]. KLF6 contributes to the pathogenesis of drug-induced hepatotoxicity [172] as well as acute liver failure [173], making it an attractive pharmacological target to alleviate hepatotoxicity. 

The present findings suggest a causative relationship between several SP/KLF members and hepatic diseases. As a next step, novel drugs can be designed to test the capacity of such interventions to attenuate disease progression. In fact, several animal and cell culture models examining the pharmacological modulation of SP/KLF members have successfully delivered promising therapeutic outcomes [83,156,159,200]. However, it remains unanswered if they can be translatable to clinical management. Several existing therapies exert their pharmacological effects by influencing transcriptional responses that are mediated by SP/KLF members. For instance, metformin, teneligliptin, and simvastatin have been found to exert some of their effects by controlling KLF family members [64,108,214]. Hence, this substantiates the importance of the exploration of avenues for drug development that will be based on the modulation of expression and activity of SP/KLF transcriptional factors. 

## Figures and Tables

**Table 1:** Summary list of the roles of SP/KLF transcription factors in various hepatic diseases. (Abbreviations: CCL4: Carbon tetrachloride, CSE: Cystathionine gamma-lyase, EMT: Epithelial-mesenchymal transition, HCC: hepatocellular carcinoma, HFD: High-fat diet, HSC: Hepatic stellate cells, HULC: Highly upregulated in liver cancer, MALAT1: Metastasis Associated Lung Adenocarcinoma Transcript 1, MMP-9: Matrix metalloproteinase 9, NAFLD: Non-alcoholic fatty liver disease, NR0B2: Nuclear receptor subfamily 0, group B, member 2, PCNA: Proliferating cell nuclear antigen, PGC-1α: Peroxisome proliferator-activated receptor-gamma coactivator (PGC)-1alpha, PON1: Paraoxonase-1, PPARα: Peroxisome proliferator-activated receptor α, Ras GRP1: RAS guanyl-releasing protein 1, RYBP: RING1 and YY1-binding protein, SREBP-1c: Sterol regulatory element binding protein-1c, TERT: Telomerase reverse transcriptase, TGF-β: Transforming growth factor β, Twist2: Twist Family BHLH Transcription Factor 2, zDHHC7: Zinc finger DHHC-type palmitoyltransferase 7). Arrow symbol ↑ denotes increase, ↓—decrease and →—connection between two events.

Transcription Factor	Insulin Resistance/NAFLD	HCC	Hepatic Fibrosis	Biliary Cholangitis	Hepatitis	Drug Induced Hepatotoxicity	Acute Liver Toxicity/Failure
SP1	↑ SREBP-1c → ↑ Hepatic lipogenesis [150] ↓ Methylation of PON1 promoter → ↑ NAFLD [151]	↑ RasGRP1, RYBP, CSE, TERT → ↑ HCC [152,153] ↑ HULC and MALAT1 lncRNAs → ↑ HCC [154,155] ↑ Bak → ↓ HCC [156]	↑ TGF-β → ↑ Fibrosis [157]	-	↑ HBV ccc DNA replication → ↑ Hepatitis B [158]	-	↑ TGF-β → ↑ Liver failure [159]
SP3	-	↑ HULC and MALAT1 lncRNAs and ↓ SP1-Bak induction →↑ HCC [154,155,156]	-	-	-	-	-
KLF2	↑ CD36 → ↑ steatohepatitis [72] Predicted link to fibrotic NASH (by IPA analysis of GSE130970) [160]	↓ Hedgehog and TGF-β → ↓ HCC [161] ↑ c-Myc → ↑ HCC [162]	-	-	-	-	↑ Activin A secretion → ↑ Hepatic damage [146]
KLF3	↓ Fam132a → ↑ Insulin resistance [53]	-	-	-	-	-	-
KLF4		↓ Slug → ↓ EMT → ↓ HCC [163]	↑ TGF-β and ↑MMP/TIMP → ↑ Fibrosis [164]	-	-	-	↑ Liver regeneration → ↓ Liver damage [165,166]
KLF5	-	↑ PI3K/Akt/Snail → ↑ EMT → ↑ HCC [167]	-	↑ Ductal regeneration → ↓ Cholangitis [168].	-	-	-
KLF6	↓miR10b → ↑ PPARα → ↑ Trb3→ ↑ Insulin resistance [76] KLF6-IVS1-27Gwt (no splicing) → ↑ KLF6-IVS1-27G>A (splice variant) →↓ NAFLD [54]	↓ PCNA, MMP-9 → ↓ HCC [169] ↓ p53 and↑ BCL-XL → ↑HCC [170]	↑ α1 collagen → ↑HSC activation → ↑ Fibrosis [171]	↓ NR0B2 → ↑ Bile duct ligation induced hepatic injury [109]	-	↑ Diclofenac induced hepatotoxicity [172]	↑ Autophagy → ↑ Hepatic damage [173]
KLF8	-	↑ Wnt/β-catenin → ↑ HCC [174]	-	-	-	-	-
KLF9	↑ CD36 → ↑ Steatohepatitis [77] ↑ PGC-1α → ↑ Hepatic gluconeogenesis → ↑ Insulin resistance [55] ↑ PGC-1α → ↑ Glucocorticoid induced diabetes [55]	-	-	-	-	-	-
KLF10	↑ Pepck → ↑ Hepatic gluconeogenesis → ↑ Insulin resistance [57] ↑ zDHHC7 → ↑ CD36 mediated FA uptake → ↑ Steatohepatitis [80] ↓ Hepatocyte apoptosis → ↓ Steatohepatitis by methionine and choline deficient diet [175]	-	↓ TGF-β → ↓Fibrosis [79]	-	-	-	-
KLF11	↑ FA oxidation → ↓ Steatohepatitis [81] ↓ *PEPCK, PGC1A* → ↓ Hepatic gluconeogenesis [61]	-	-	-	-	-	-
KLF13	↑ Expression → ↑ NAFLD in humans [23]	↑ Cholesterol synthesis → ↑ HCC [176]	-	-	-	-	-
KLF14	↑ PGC-1α → ↑ Hepatic gluconeogenesis → ↑ Insulin resistance [59] ↑ PI3K/Akt → ↑ Hepatic insulin sensitivity [82] Gene polymorphism in *KLF14* → ↑ type II diabetes [177]	-	-	-	-	-	-
KLF15	↑Twist2 → ↓ Steatohepatitis [178] ↑ Recruitment of RIP40 corepressor to the SREBF-1 promoter → ↓ Hepatic lipogenesis [86] ↑ ER stress → ↑ Insulin resistance [85]	-	-	-	↑ Core and surface genes expression → ↑Hepatitis B [179]	-	↓ p38 MAPK/ERK1/2 → ↓ Hepatic injury [180]
KLF16	↑ PPARα → ↑ FA oxidation → ↓ Steatohepatitis and NAFLD [84]	-	-	-	-	-	-

## Data Availability

Not applicable.

## Abbreviations

α-SMA	Alpha smooth muscle actin
ABCA1	ATP binding cassette subfamily A member 1
ABCG1	ATP binding cassette subfamily G member 1
ACC	Acetyl-CoA carboxylase
AG	Alpha glucosidase
ALT1	Alanine transaminase
AGPAT1	1-acylglycerol-3-phosphate O-acyltransferase 1
Akt	AKT serine/threonine kinase 1
Apo A-I	Apolipoprotein A-I
Apo A-IV	Apolipoprotein A-IV
AMPK	Adenosine monophosphate activated protein kinase
ApoE	Apolipoprotein E
AP-1	Activator protein 1
AhR	Aryl hydrocarbon receptor
Atg7	Autophagy related 7 gene
ATP7B	ATPase copper transporting beta
Btd	Buttonhead box domain
Bmal1	basic helix-loop-helix ARNT Like 1
BA	Bile acids
Becn1	Beclin1
c/EBP	CCAAT-enhancer-binding proteins
ChREBP	Carbohydrate response element binding protein
CtBP	C-terminal-binding protein
CFU-E	Colony forming unit-Erythroid
CFU-MK	Colony forming unit-Megakaryocyte
CXCR4	C-X-C chemokine receptor type 4
CPS1	Carbamoyl-phosphate synthase 1
CYP450	Cytochrome P450
CYP1A1	Cytochrome P450 family 1 subfamily A member 1
CYP1A2	Cytochrome P450 family 1 subfamily A member 2
CYP2B10	Cytochrome P450, family 2, subfamily b, polypeptide 10
CYP2D6	Cytochrome P450 family 2 subfamily D member 6
CYP2E1	Cytochrome P450 family 2 subfamily E member 1
CYP3A4	Cytochrome P450 family 3 subfamily A member 4
CY4A10	Cytochrome P450, family 4, subfamily a, polypeptide 10
CYP4A14	Cytochrome P450, family 4, subfamily a, polypeptide 14
CYP7A1	Cytochrome P450 family 7 subfamily A member 1
CYP27A1	Cytochrome P450 family 27 subfamily A member 1
CYP13A8	*C.elegans* cytochrome P450 family 13 subfamily A member 8
CYP13A11	*C.elegans* cytochrome P450 family 13 subfamily A member 11
CYP33C9	*C.elegans* cytochrome P450 family 33 subfamily C member 9
CYP37A1	*C.elegans* cytochrome P450 family 37 subfamily A member 1
cAMP	3',5'-cyclic adenosine monophosphate
CREB	cAMP response element-binding protein
CCL4	Carbon tetrachloride
ChIP	Chromatin immunoprecipitation
CPT1A	Carnitine palmitoyltransferase 1A
CETP	Cholesteryl ester transfer protein
CA	Cholicacid
CDCA	Chenodeoxy cholicacid
CDX2	Caudal-type homeobox 2
c-MYC	MYC proto-oncogene
CSE	Cystathionine gamma-lyase
ccc DNA	Covalently closed circular DNA
DBP	D site of albumin promoter (albumin D-box) binding protein
DEX	Dexamethasone
DGAT1	Diacylglycerol O-acyltransferase 1
DDC	3,5-diethoxycarbonyl-1,4-dihydrocollidine
ESLD	End stage liver disease
EKLF	Erythroid Krüppel-like factor
EMSA	Electrophoretic mobility shift assay
eNOS	Endothelial nitric oxide synthase
ERG	ETS related gene
ETF	ETF transcription factor
E2F	E2F family of transcription factors
EMT	Epithelial–mesenchymal transition
ERK	Extracellular signal-regulated kinases
FOXA2	Forkhead Box A2
FOXO1	Forkhead box O1
FOXO3a	Forkhead box O3
Fam132a	Adipose-derived insulin-sensitizing factor; Adipolin
FGF15	Fibroblast growth factor 15
FGF21	Fibroblast growth factor 21
FASN	Fatty acid synthase
FBW7α	F-box and WD repeat domain containing 7
FATP	Fatty acid transport protein
FXR	Farnesoid X receptor
GATA1	GATA-binding factor 1
GATA4	GATA binding factor 4
GATA6	GATA-binding factor 6
GLUT2	Glucose transporter 2
GCK	Glucokinase
G6PC	Glucose 6-phosphatase
GPAT1	Glycerol-3-phosphate acyltransferase, mitochondrial
GSK-3β	Glycogen synthase kinase 3 beta
Gpbar1	G protein-coupled bile acid receptor 1
GST	Glutathione-s-transferase
HCC	Hepatocellular carcinoma
Hhex	Hematopoietically expressed homeobox
HNF4	Hepatocyte nuclear factor 4
HGF	Hepatocyte growth factor
HNF3β	Hepatocyte nuclear factor 3-beta
HFD	High fat diet
HDL	High density lipoprotein
HPD	4-hydroxyphenylpyruvate dioxygenase
HUVEC	Human umbilical vein endothelial cells
HULC	Hepatocellular carcinoma up-regulated long non-coding RNA
HBV	Hepatitis B virus
HFE	Homeostatic iron regulator
InsR	Insulin receptor
IRS-1	Insulin receptor substrate 1
iPSCs	Induced pluripotent stem cells
KLFs	Krüppel-like family of transcription factors
LXR	The liver X receptor
LDL	Low density lipoprotein
LCAT	Lecithin-cholesterol acyltransferase
LncRNA	Long noncoding RNA
MCAD	Medium-chain acyl-CoA dehydrogenase
MAPK	Mitogen-activated protein kinases
MALAT1	Metastasis associated lung adenocarcinoma transcript 1
MMP-1	Matrix metallopeptidase 1
MMP-9	Matrix metallopeptidase 9
NASH	Nonalcoholic steatohepatitis
NAFLD	Nonalcoholic fatty liver disease
NEFA	Non-esterified fatty acids
NAG	N-Acetylglutamate
NAGS	N-Acetylglutamate Synthetase
NR0B2	Nuclear receptor subfamily 0 group B member 2
NF-κB	Nuclear factor kappa B
NCOA3	Nuclear receptor coactivator 3
OSM	Oncostatin M
OTC	Ornithine transcarbamylase
OCT-4	Octamer-binding transcription factor 4
ODN	Oligodeoxynucleotides
PARP1	Poly ADP ribose polymerase 1
PGC-1α	Peroxisome proliferator-activated receptor-gamma coactivator (PGC)-1alpha
PPARα	Peroxisome proliferator-activated receptor alpha
PPARγ	Peroxisome proliferator-activated receptor gamma
PCK1	Phosphoenolpyruvate carboxykinase 1
PEPCK	Phosphoenolpyruvate carboxykinase
PI3K	Phosphoinositide 3-kinase
PRODH	Proline dehydrogenase
PSC	Primary sclerosing cholangitis
PAI-I	Plasminogen activator inhibitor 1
PON1	Paraoxonase 1
PCNA	Proliferating cell nuclear antigen
P53	Tumor protein P53
PDGF-β	Platelet derived growth factor-B
PXR	Pregnane X receptor
RORα	Retinoid-related orphan receptor alfa
RasGRP1	RAS guanyl releasing protein 1
RYBP	RING1 and YY1 binding protein
SREBF1	Sterol regulatory element binding transcription factor 1
SREBP-1C	Sterol regulatory element-binding protein 1
SP	Specificity protein
SOX17	SRY-Box transcription factor 17
SR-B1	The scavenger receptor, class B type 1
SCD1	Stearoyl-Coenzyme A desaturase 1
SSW	Sweetened sucrose water
SULT1A1	Sulfotransferase family 1A member 1
SULT2B1B	Sulfotransferase family 2B member 1
SOX-2	(Sex determining region Y)-box 2
SMAD3	SMAD family member 3
TAT	Tyrosine aminotransferase
TGs	Triglycerides
TDO2	Tryptophan 2,3-Dioxygenase
TGR-5	Takeda G protein-coupled receptor 5
TSS	Transcriptional start site
TM	Thrombomodulin
TF	Tissue factor
TGF-β	Transforming growth factor beta
TERT	Telomerase reverse transcriptase
TIMP-1	Tissue inhibitor matrix metalloproteinase 1
TNF-α	Tumor necrosis factor α
UPR	Unfolded protein response
UGT1A8	UDP glucuronosyltransferase family 1 member A8
UGT1A9	UDP Glucuronosyltransferase Family 1 Member A9
UGT1A10	UDP Glucuronosyltransferase Family 1 Member A10
VCAM-1VLDL	Vascular cell adhesion molecule-1Very low-density lipoprotein
WD	Wilsons disease
WNT	Proto-oncogene Wnt
WAT	White adipose tissue
XRE	Xenobiotic response element
YY1	Yin Yang 1
ZDHHC7	Zinc finger DHHC domain-containing protein 7
ZBP-89	Zinc-finger binding protein-89
